# Benefits of clean air for school children's vision health

**DOI:** 10.1093/pnasnexus/pgaf279

**Published:** 2025-09-23

**Authors:** Xi Chen, Yuqing Dai, Ruihua Wei, Bei Du, Congchao Lu, A Robert MacKenzie, Nai-jun Tang, Zongbo Shi, Hua Yan

**Affiliations:** Department of Occupational and Environmental Health, School of Public Health, Tianjin Medical University, No. 22 Qixiangtai Road, Heping District, Tianjin 300070, China; School of Geography, Earth and Environmental Science, University of Birmingham, Birmingham B15 2TT, United Kingdom; Tianjin Key Laboratory of Retinal Functions and Diseases, Tianjin Branch of National Clinical Research Center for Ocular Disease, Eye Institute and School of Optometry, Tianjin Medical University Eye Hospital, No. 251 Fukang Road, Nankai District, Tianjin 300384, China; Tianjin Key Laboratory of Retinal Functions and Diseases, Tianjin Branch of National Clinical Research Center for Ocular Disease, Eye Institute and School of Optometry, Tianjin Medical University Eye Hospital, No. 251 Fukang Road, Nankai District, Tianjin 300384, China; Department of Occupational and Environmental Health, School of Public Health, Tianjin Medical University, No. 22 Qixiangtai Road, Heping District, Tianjin 300070, China; School of Geography, Earth and Environmental Science, University of Birmingham, Birmingham B15 2TT, United Kingdom; Department of Occupational and Environmental Health, School of Public Health, Tianjin Medical University, No. 22 Qixiangtai Road, Heping District, Tianjin 300070, China; School of Geography, Earth and Environmental Science, University of Birmingham, Birmingham B15 2TT, United Kingdom; Department of Ophthalmology, Laboratory of Molecular Ophthalmology and Tianjin Key Laboratory of Ocular Trauma, Ministry of Education International Joint Laboratory of Ocular Diseases, Tianjin Institute of Eye Health and Eye Diseases, China-UK “Belt and Road” Ophthalmology Joint Laboratory, Tianjin Medical University General Hospital, Tianjin 300052, China

**Keywords:** air quality, uncorrected visual acuity, myopia, environmental health, automated machine learning

## Abstract

Myopia has become a significant public health concern among school-aged children in East Asia. A growing body of evidence acknowledges the multifactorial nature of myopia, including genetic susceptibility, lifestyle habits, and environmental influences. However, the specific role of air quality on vision remains poorly understood due to many confounding variables. Here, we applied an explainable machine learning framework with a large multifactorial cohort to identify key drivers of uncorrected visual acuity and to quantify the potential vision benefits of cleaner air in nearly 30,000 school-aged children. We show that, after controlling for potential confounders, lower ambient nitrogen dioxide and fine particles levels are independently associated with better vision. Primary school students and children with mild-to-moderate myopia benefit more from cleaner air than highly myopic or senior school students. These findings reinforce the emerging view that air pollution plays a significant and modifiable role in visual development. Importantly, our results uniquely indicate that early interventions to reduce air pollution exposure for younger children could yield greater benefits.

Significance Statement Myopia is becoming a global public health issue, particularly in East Asia, where school-aged children are experiencing high prevalence rates. While genetic factors are well-documented, this study highlights the significant role of environmental factors, such as air pollution, in visual health. By analyzing data from nearly 30,000 children, we found that better air quality was linked to improved uncorrected visual acuity, especially in younger children. This suggests that reducing air pollution exposure in early years could help slow myopia progression, highlighting the importance of early interventions targeting both environmental and lifestyle factors.

## Introduction

Myopia, commonly known as short- or near-sightedness, has emerged as a pressing public health concern globally. In East Asia its prevalence among school-leavers has reached 80–90%, and 10–20% of those students already have high myopia (1). The etiology of myopia is multifactorial, and its development is driven by a combination of environmental, behavioral, and genetic factors (2). Well-established risk factors include environmental stressors linked to modern urban lifestyles (e.g. intensive reading or screen use) (3, 4), genetic predisposition (e.g. having myopic parents), and behavioral habits such as increased educational pressures that reduce outdoor activities and exposure to natural light.

Recent research has shown ambient air pollution as a potential contributor to myopia development among children. Epidemiological studies indicate that children in regions with worse air quality tend to have poorer visual outcomes. For example, an area-level analysis in China found that communities with higher fine particles (PM_2.5_) concentrations had a significantly greater prevalence of reduced visual acuity in youth (5). Some studies suggested that air pollution is an important exacerbating agent that may elevate oxidative stress and contribute to the pathogenesis of ocular surface inflammation (6–8), which indicates that chronic pollution exposure may elevate myopia risk. Improvements in ambient air quality (i.e. cleaner air) should theoretically benefit visual acuity, but direct evidence for such positive effects is limited. A major challenge in quantifying these benefits is the need to account for confounding influences from socioeconomic, genetic, or lifestyle factors that also affect vision.

Traditional linear or logistic regression struggle to untangle the complex patterns in large health datasets. In the context of children's vision, factors such as genetics, educational environment, and daily activities likely interact in nonlinear ways to influence uncorrected visual acuity (UCVA). Traditional models require manual specification of interaction terms and often assume additive or linear effects, which risks oversimplifying reality (2). Automated machine learning (AutoML) offers a more flexible analytical approach that can handle multiple variables and automatically model interactions or nonlinear influences without requiring a priori specification of those relationships. They have been applied in various environmental health studies, such as identifying key dietary pollutant sources, modeling joint exposure to air pollution and temperature extremes, predicting disease spread using air quality data, and evaluating clinical outcomes influenced by environmental conditions (9–13). Additionally, previous work reported that machine learning approaches can identify risk factors not previously highlighted and achieve higher predictive accuracy than logistic regression when analyzing complex epidemiological data (14). Therefore, applying AutoML enables us to re-evaluate known risk factors under a unified, interaction-aware model and to identify patterns that traditional regression might neglect. Detailed demographic and behavioral information on children is scarce, and few analytical frameworks can integrate the variables that do exist. We therefore need studies that assess air pollution together with the full range of myopia risk factors to clarify each factor's individual and joint effects.

Here, we aim to apply an explainable AutoML framework to a large, multifactorial dataset to (i) identify the key drivers of childhood myopia, (ii) quantify the vision benefits achievable through cleaner air, and (iii) reveal age- and severity-specific differences in risk-factor profiles. The dataset used in this analysis includes information on familial myopia history (genetic predisposition), academic pressure and schooling characteristics (educational factors), daily activity patterns such as outdoor exposure or screen time (lifestyle factors), and environmental factors including air quality. To our knowledge, this is the first study to incorporate these detailed variables for comprehensive adjustment of potential confounders in modeling myopia risk. This holistic approach and the use of AutoML enable us to identify the impact of air quality on children's vision outcomes while accounting for the effects of other risk factors, which was not previously possible.

## Results

### Participant characteristics and environmental exposure

**Fig. 1.**
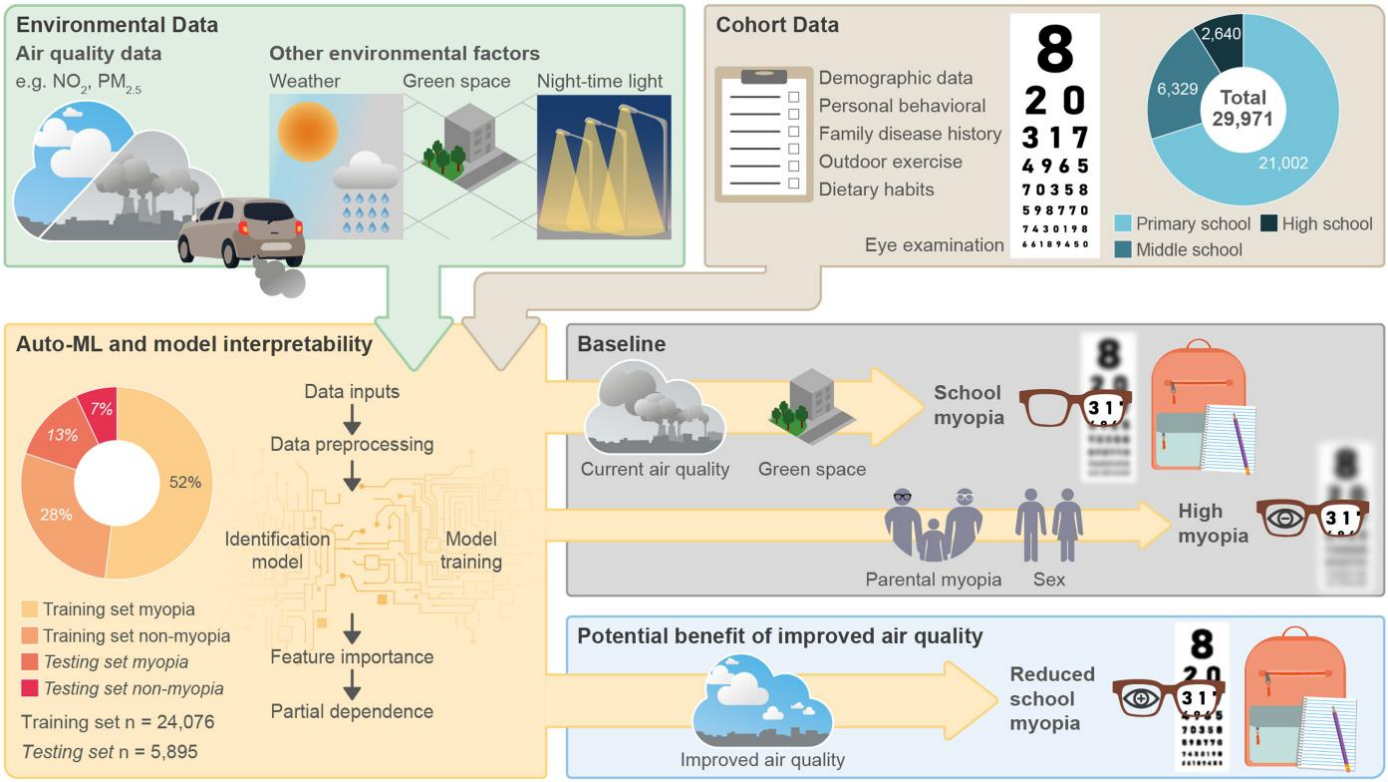
Framework for analyzing visual health and environmental impact. The diagram presents the integration of cohort and environmental data to evaluate the impact on visual health. It includes the collection of air quality data and other environmental factors like weather, green space, and nighttime light, alongside personal data such as demographics, behavior, family disease history, outdoor exercises, dietary habits, and eye examinations. The AutoML process is detailed, showing data inputs, preprocessing, model training, feature importance, and partial dependence analysis. The potential benefits of improved air quality on reducing school myopia are highlighted, demonstrating the significance of environmental improvements on visual acuity.

Figure 1 shows the study design. This study enrolled 29,971 participants (mean age 10.4 ± 2.8 years) from 16 districts of Tianjin, comprising 15,569 (51.9%) males and 14,402 (48.1%) females based on sex assigned at birth. Students living in urban areas represented 84.7% (25,392), with the rest (4,579) from rural areas. Myopia prevalence was 53.2% on average, with median and interquartile ranges of 4.90 (4.50, 5.00) for UCVA and −0.75 (−2.50, 0.00) for spherical equivalent for both eyes.

Sleep duration varies by grade: 9.8% of primary school students slept ≥10 h, 4.0% of junior high-school students slept ≥9 h, and 12.9% of senior high-school students slept ≥8 h. Additionally, 11.0% of all students reported sleeping with lights on. Parental myopia prevalence was 64.5% with one myopic parent. Dietary habits showed that 78.8% consumed 4–6 g of salt per day, and 81.9% ate desserts ≤3 times per week. Median values (and interquartile range) of exposure to 2-year average air pollutants were 38.6 (38.0–39.0) μg m^−3^ for PM_2.5_, and 33.6 (33.0–35.3) μg m^−3^ for nitrogen dioxide (NO_2_). Median normalized difference vegetation index (NDVI) was 0.23 (0.21, 0.28), and median nighttime lighting was 52.6 nW cm^−2^ per steradian (13.9, 83.4). Participant characteristics are summarized in Table 1. Flowchart of participant selection process is provided in Fig. S1.

**Table 1.** Comparison of demographic and clinical characteristics and outcomes between myopia and nonmyopia.

Variables	Total (*n* = 29,971)	Myopia (*n* = 15,948)	Nonmyopia (*n* = 14,023)	*P* value
UCVA, P_50_ (P_25_, P_75_)	4.8 (4.4, 5.0)	4.5 (4.2, 4.7)	5.0 (4.90, 5.10)	<0.001
Spherical equivalent refraction, P_50_ (P_25_, P_75_)	−2.75 (−1.13, −0.25)	−0.25 (−4.0, −1.38)	−0.25 (−0.50, 0.13)	<0.001
Sex, *n* (%)				<0.001
Female	14,402 (48.1)	8,027 (50.3)	6,375 (45.5)	
Male	15,569 (51.9)	7,921 (49.7)	7,648 (54.5)	
Parental myopia, *n* (%)				<0.001
Neither	10,642 (35.5)	5,108 (32.0)	5,534 (45.5)	
Either	19,329 (64.5)	10,840 (68.0)	7,648 (54.5)	
BMI^a^, P_50_ (P_25_, P_75_)	17.6 (15.2, 20.3)	17.9 (15.6, 20.5)	17.1 (14.9, 20.0)	<0.001
Level of education, *n* (%)				<0.001
Primary school	21,002 (70.1)	8,723 (54.7)	12,279 (87.6)	
Middle school	6,329 (21.1)	4,964 (31.1)	1,365 (9.7)	
High school	2,640 (8.8)	2,261 (14.2)	379 (2.7)	
Mode of commuting to school, *n* (%)				<0.001
Nonmotorized transport	17,199 (57.4)	8,808 (55.2)	8,391 (59.8)	
Private car	10,362 (34.6)	5,506 (34.5)	4,856 (34.6)	
Public transport	2,147 (7.2)	1,411 (8.8)	736 (5.2)	
Residential	263 (0.9)	223 (1.4)	40 (0.3)	
Seat rows, *n* (%)				0.018
1–3 rows	13,098 (43.7)	7,079 (44.4)	6,019 (42.9)	
4–6 rows	13,082 (43.6)	6,910 (43.3)	6,172 (44.0)	
7–10 rows	3,791 (12.6)	1,959 (12.3)	1,823 (13.1)	
Time of homework^b^, P_50_ (P_25_, P_75_)	1.0 (1.0, 2.0)	1.0 (1.0, 2.0)	1.0 (1.0, 2.0)	<0.001
Time of outdoor activities (h)^c^, *n* (%)				<0.001
<1	7,994 (26.7)	4,860 (30.5)	3,134 (22.3)	
1–2	16,151 (53.9)	8,376 (52.5)	7,775 (55.4)	
2–3	4,387 (14.6)	2,028 (12.7)	2,359 (16.8)	
>3	1,439 (4.8)	684 (4.3)	755 (5.4)	
Reading distance, *n* (%)				<0.001
Less than a foot	6,208 (20.7)	3,608 (22.6)	2,600 (18.5)	
Greater than a foot	23,763 (79.3)	12,340 (77.4)	11,423 (81.5)	
Rubbing eyes, *n* (%)				<0.001
Never	4,272 (14.3)	1,808 (11.3)	2,464 (17.6)	
Sometimes	23,227 (77.5)	12,562 (78.8)	10,665 (76.1)	
Frequent	2,250 (7.5)	1,440 (9.0)	810 (5.8)	
Always	222 (0.7)	138 (0.9)	84 (0.6)	
Ball games, *n* (%)				<0.001
Never	8,570 (28.6)	4,765 (29.9)	3,805 (27.1)	
1 kind	13,824 (46.1)	7,332 (46.0)	6,492 (46.3)	
2 kinds	5,519 (18.4)	2,809 (17.6)	2,710 (19.3)	
3 kinds	1,646 (5.5)	825 (5.2)	821 (5.9)	
4 kinds	331 (1.1)	170 (1.1)	161 (1.1)	
5 kinds	81 (0.3)	47 (0.3)	34 (0.2)	
Sleep duration^d^, P_50_ (P_25_, P_75_)	8.50 (9.00, 9.67)	9.00 (8.33, 9.50)	9.45 (9.00, 9.83)	<0.001
Frequency of going to green spaces, *n* (%)				<0.001
Never	1,309 (4.4)	848 (5.3)	461 (3.3)	
Once every few month	6,100 (20.4)	3,692 (23.2)	2,408 (17.2)	
More than 1 time per month	17,203 (57.4)	8,926 (56.0)	8,277 (59.0)	
Almost every day	5,359 (17.9)	2,482 (15.6)	2,877 (20.5)	
Frequency of soft drinks, *n* (%)				<0.001
Hardly ever	11,136 (37.2)	5,437 (34.1)	5,699 (40.6)	
<1 time/week	11,034 (36.8)	5,920 (37.1)	5,114 (36.5)	
2–3 times/week	61.89 (20.6)	3,572 (22.4)	2,617 (18.7)	
4–6 times/week	921 (3.1)	578 (3.6)	343 (2.4)	
1 time/day	462 (1.5)	286 (1.8)	176 (1.3)	
2 or more times/day	229 (0.8)	155 (1.0)	74 (0.5)	
Daily dietary salt intake, *n* (%)				<0.001
<4 g	4,724 (15.8)	2,284 (14.3)	2,440 (17.4)	
4–6 g	23,630 (78.8)	12,648 (79.3)	10,982 (78.3)	
>6 g	1,617 (5.4)	1,016 (6.4)	601 (4.3)	
Frequency of seafood intake, *n* (%)				0.005
Hardly ever	3,612 (12.1)	2,021 (12.7)	1,591 (11.3)	
<1 time/week	8,923 (29.8)	4,761 (29.9)	4,162 (29.7)	
2–3 times/week	14,363 (47.9)	7,542 (47.3)	6,821 (48.6)	
4–6 times/week	2,232 (7.4)	1,164 (7.3)	1,068 (7.6)	
Once a day	586 (2.0)	314 (2.0)	272 (1.9)	
More than 2 times a day	255 (0.9)	146 (0.9)	109 (0.8)	
Nightlight, P_50_ (P_25_, P_75_)	52.63 (13.92, 83.44)	52.63 (13.92, 83.44)	52.63 (13.92, 83.44)	<0.001
NDVI^e^, P_50_ (P_25_, P_75_)	0.24 (0.21, 0.28)	0.24 (0.21, 0.28)	0.24 (0.21, 0.28)	<0.001
PM_2.5_, P_50_ (P_25_, P_75_)	38.50 (37.25, 38.95)	38.55 (37.50, 39.00)	38.45 (37.10, 38.90)	<0.001
NO_2,_ P_50_ (P_25_, P_75_)	36.45 (34.20, 37.50)	36.60 (34.50, 37.50)	35.80 (33.90, 37.50)	<0.001

UCVA, uncorrected visual acuity; BMI, body mass index; NDVI, normalized difference vegetation index (NDVI); PM_2.5_, particulate matter smaller than 2.5 µm; NO_2_, nitrogen dioxide.

^a^BMI was missing in 503 cases in the myopia and 627 cases in the nonmyopia.

^b^Homework hours were missing in 867 cases in the myopia and 303 cases in the nonmyopia.

^c^Outdoor hours were missing in 684 cases in the myopia and 755 cases in the nonmyopia.

^d^Sleep duration was missing in 651 cases in the myopia and 319 cases in the nonmyopia.

^e^NDVI was missing in 43 cases in the myopia and 3 cases in the nonmyopia.

### Identification of key drivers of UCVA

The data collected in the eye health screening were used to develop 30 machine learning models to predict UCVA in school-aged children (Fig. 1). The discriminative performance of all models is listed in Table S1. Among these models, the gradient boosting machines (GBM) model had the best predictive power for UCVA with a root mean square error (RMSE) of 0.303, closely followed by the extreme gradient boosting (XGBoost) model with an RMSE of 0.304. Thus, the GBM model was selected as the final predictive model. Table S2 showed the demographic and clinical characteristics between the training and testing sets.

**Fig. 2.**
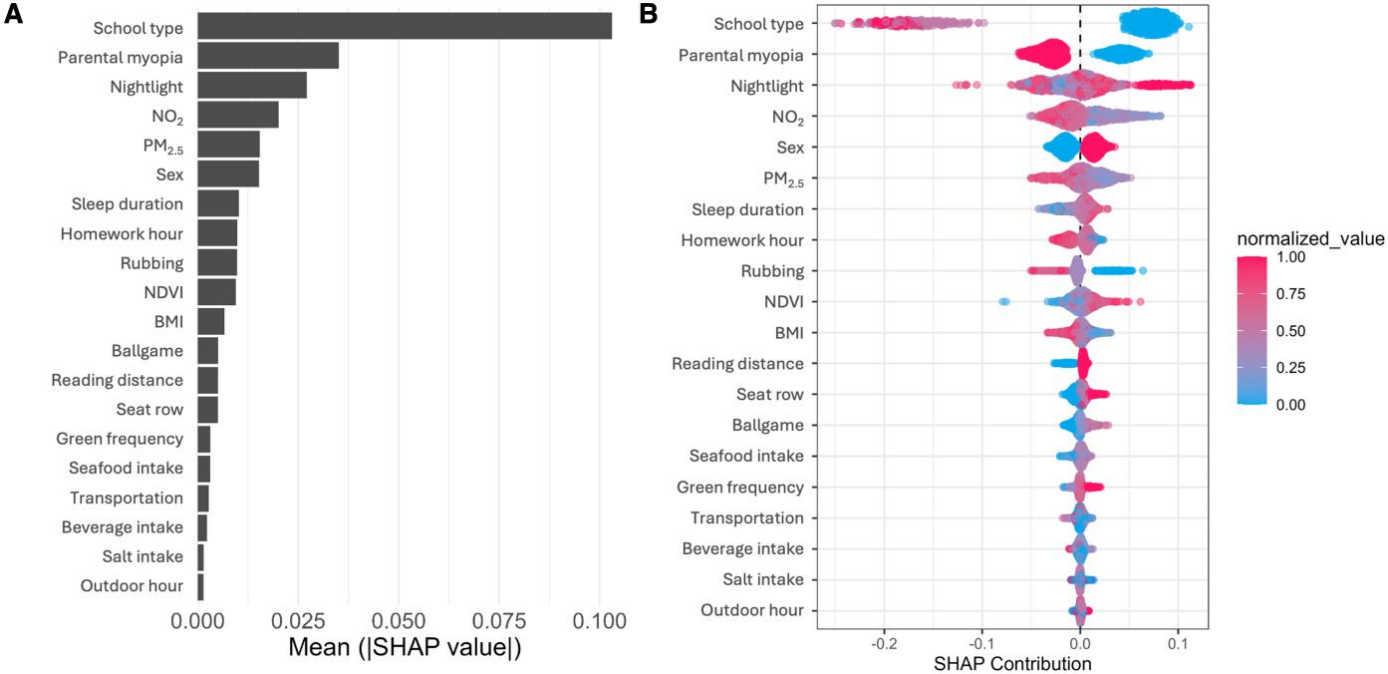
Feature importance and SHAP values for UCVA prediction. A) The bar chart presents the mean of absolute SHAP values for features used in the model predicting uncorrected visual acuity (UCVA), ranked by importance. “School type” is the most influential feature, followed by “parental myopia,” “nightlight,” “NO_2_,” and “PM_2.5_.” Other significant features include “sex,” “sleep duration,” “homework hour,” “rubbing,” “NDVI,” “BMI,” “ballgame,” “reading distance,” “seat row,” “green frequency,” “seafood intake,” “transportation,” “beverage intake,” “salt intake,” and “outdoor hour.” B) The beeswarm plot illustrates the distribution of SHAP values for each feature across the dataset, where a negative SHAP value indicates a tendency to make eyesight worse. Each point represents an instance (i.e. a member of the cohort), with its particular SHAP value for a feature given by the *x*-axis. The point shading denotes the relative magnitude of the feature value. The density and spread of dots reflect the magnitude and interaction effects of each feature. For example, higher absolute values of “school type” and “parental myopia” significantly contribute to UCVA predictions, as shown by the dense and widely spread red dots. Dot colors are min–max scaled solely for visualization, categorical predictors entered the model as factors, not numeric variables.

**Fig. 3.**
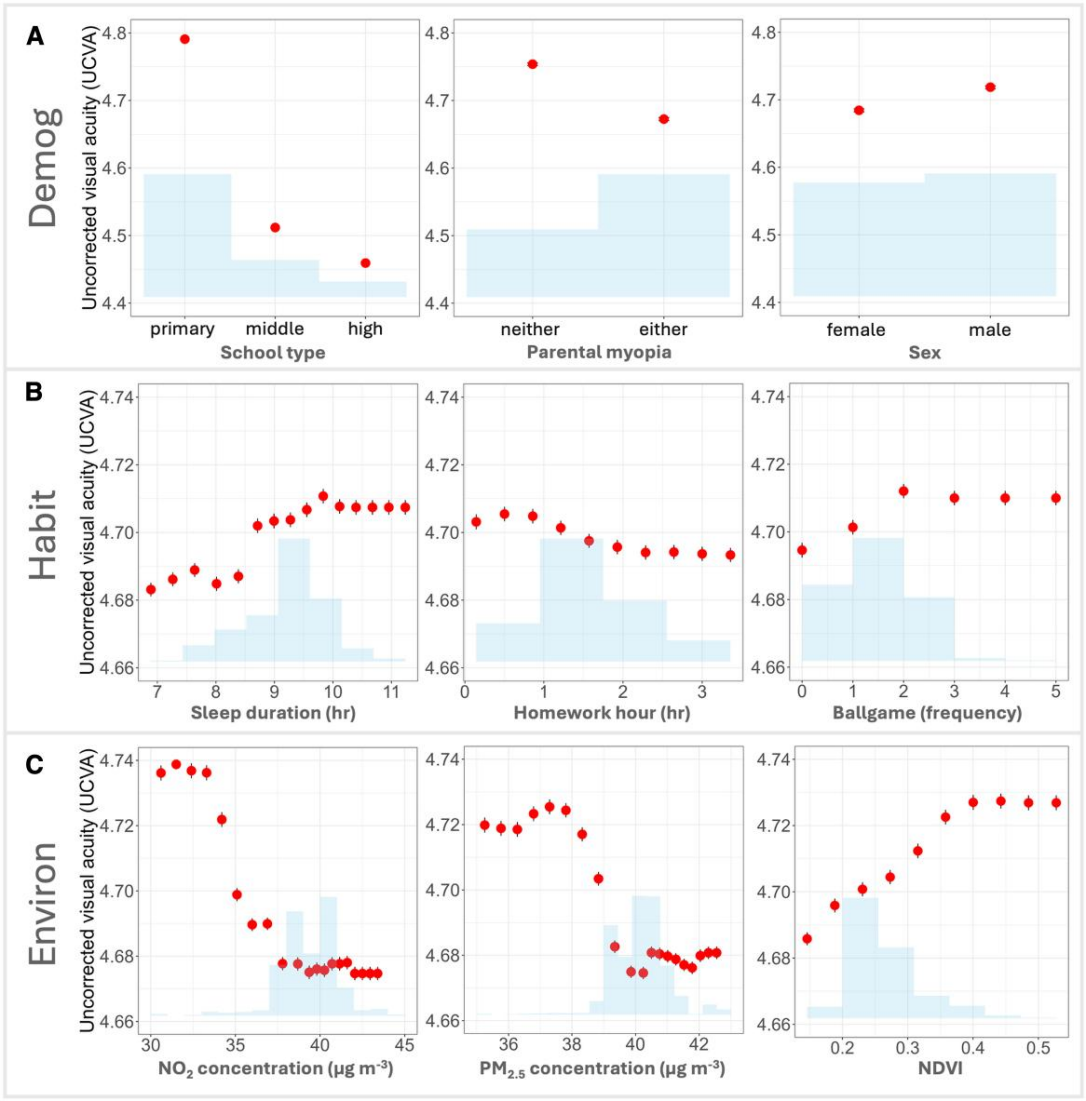
Partial dependence plots for key features affecting UCVA. Partial dependence plots showing how each feature relates to UCVA. The plots are categorized into three groups: A) Demographic factors (Demog): school type, parental myopia, and sex. B) Lifestyle habits (Habit): sleep duration, homework hours, and participation in ball games. C) Environmental factors (Environ): NO_2_ concentration, PM_2.5_ concentration, and normalized difference vegetation index (NDVI). The *x*-axis represents the feature of interest, and the *y*-axis represents UCVA, with shaded areas indicating CI or data distribution.

We used SHapley Additive exPlanations (SHAP) values with the selected GBM model to investigate the key factors affecting UCVA of school students. Figure 2A shows the mean absolute SHAP values (i.e. relative importance ranking) for all predictive features. The most influential factors associated with the changes of UCVA among school-aged children include school type (i.e. primary, middle, or high school, with mean absolute SHAP ∼0.104), parental myopia (∼0.035), nighttime light (a simple spatial proxy for socioeconomic status, ∼0.028), NO_2_ levels (∼0.020), and PM_2.5_ levels (∼0.015). Figure 2B presents a “beeswarm” plot for the top features, illustrating the distribution of SHAP values for individual observations arranged by feature importance, with point color representing each feature's magnitude and thereby showing how higher or lower values shift UCVA predictions across the student population. School type and parental myopia are identified as two highly significant factors, with attendance at primary school (blue spots) generally with higher UCVA, whereas attendance at middle and high school (red and pink spots) have negative UCVA outcomes. Environmental factors such as NO_2_ and PM_2.5_ levels, and greenspace (NDVI) exhibited similarly coherent patterns. Heavier pollution is associated with negative SHAP values, whereas greener surroundings were linked to better UCVA. We grouped key features into three main groups, including demographic factors (Demog), lifestyle habits (Habit), and environmental factors (Environ) and further investigated their influences (Fig. 3). This partial dependence analysis aligns and extends the patterns observed in Fig. 2B. Taken together, the findings indicate that myopia arises from multiple influences, whose effects interact with one another, sometimes amplifying and sometimes offsetting their individual impacts. Examining any single factor in the model on its own could misrepresent its true weight, which is critical for accurately understanding and mitigating myopia development.

### Subgroup analysis by myopia type and school level

**Fig. 4.**
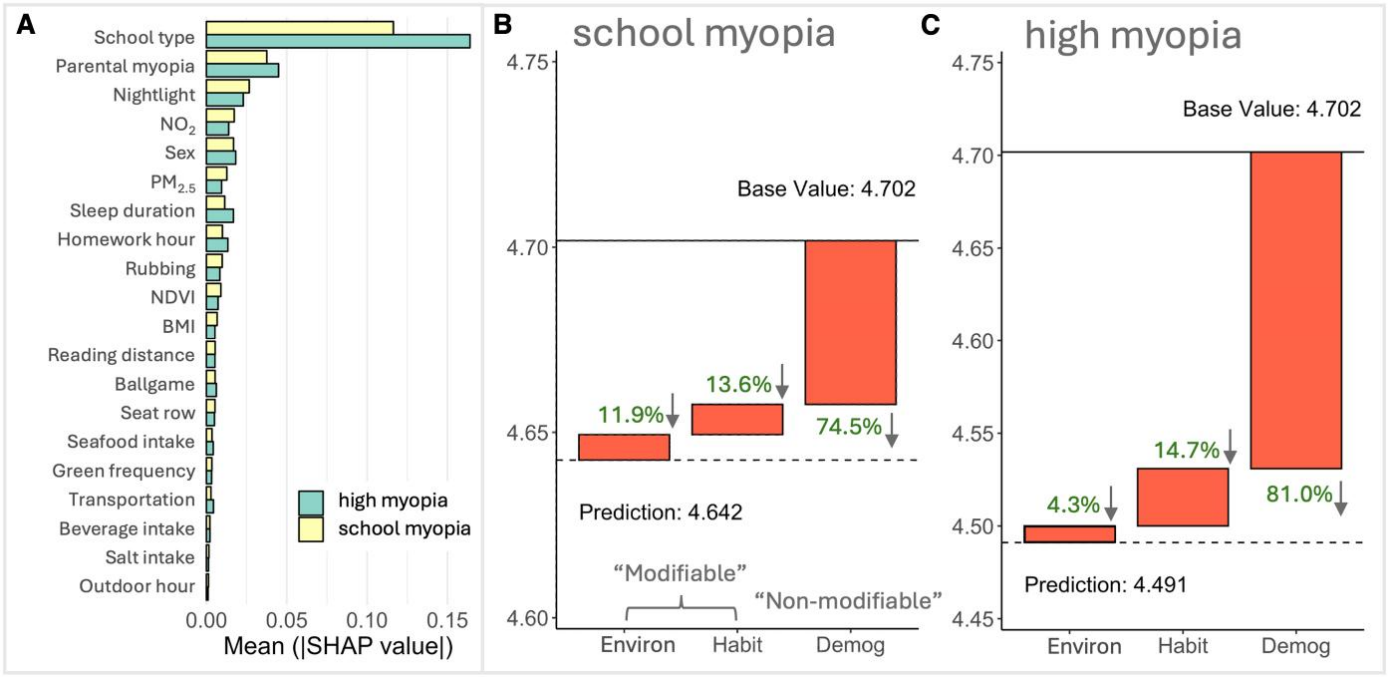
The relative importance and impact of predictive features on UCVA in students with school and high myopia. A) Bar graphs indicate the average impact of different features on UCVA within high and school myopia student groups. Waterfall charts show each feature's cumulative effect on UCVA for B) students with school myopia and C) with high myopia. The base value is the average predicted UCVA across all students, blue bars (with up arrow) indicate positive contributions (features that improve UCVA), while orange bars (with down arrow) indicate negative contributions. Arrows indicate whether the overall impact of each factor on students’ UCVA is positive (up) or negative (down).

**Fig. 5.**
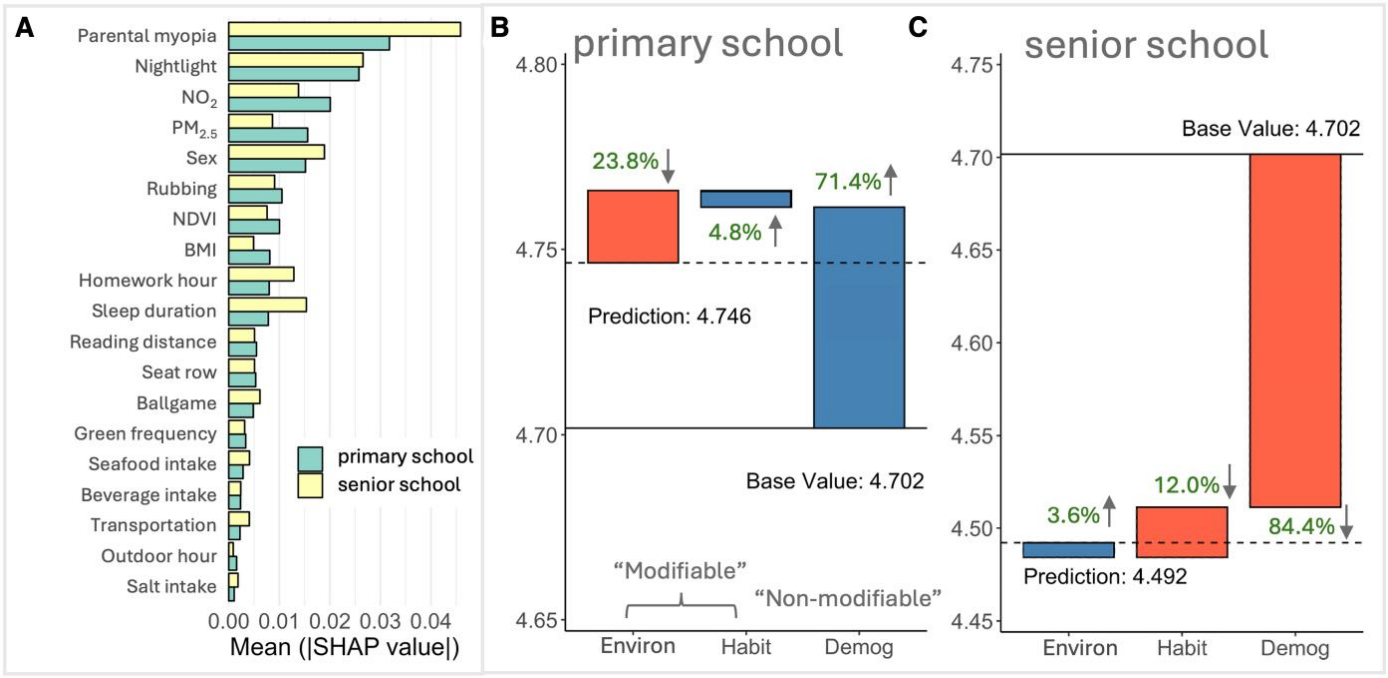
The relative importance and impact of predictive features on UCVA in primary and senior (middle and high) school students. A) Bar graphs indicate the average impact of different features on UCVA within primary and senior school student groups. Waterfall charts illustrate how each feature contributes cumulatively to UCVA for B) primary school students and C) middle- and high-school students. The base value is the average predicted UCVA across all students, blue bars (with up arrow) indicate positive contributions (features that improve UCVA), while orange bars (with down arrow) indicate negative contributions. Arrows indicate whether the overall impact of each factor on students’ UCVA is positive (up) or negative (down).

We conducted a subgroup analysis using “mean SHAP” and local SHAP explanations to examine whether the contributions of these risk factors differed by myopia severity and school level. Comparison of demographic and clinical characteristics across subgroups is shown in Tables S3 and S4. Our results show obvious differences in relative importance of input features among different subgroups (Figs. 4 and 5). Details of the contributions of each feature are provided in Figs. S2 and S3.

Among children with high myopia (severe refractive error >−6.00 diopters), UCVA was mainly explained by nonmodifiable factors. Demographic/genetic influences (e.g. parental myopia, sex, and age) accounted for about 81.0% of the total SHAP-predicted UCVA variation in this subgroup (Fig. 4C), whereas modifiable environmental factors contributed only 4.3% and habits 14.7%. In contrast, in children with less severe “school myopia” (known as juvenile-onset myopia, typical mild/moderate myopia during the school-age years), modifiable factors played a larger role. That is, environmental factors explained 11.9% of their UCVA variation, and behavioral habits another 13.6% (Fig. 4B). Thus, roughly one-quarter of the risk in school myopia was attributable to modifiable influences, nearly double the relative contribution seen in high myopia. Correspondingly, the importance ranking of specific features shifted between these groups (Fig. 4A). School type had a great impact in high myopia (mean absolute SHAP ∼0.16 versus ∼0.11 in school myopia), reflecting influences from academic pressures and also the trend of high myopia prevalence increasing with age. By comparison, NO_2_ was a more influential predictor in school myopia (mean absolute SHAP ∼0.018) than in high myopia (∼0.014), and PM_2.5_ contributed ∼0.013 versus ∼0.009 of mean absolute SHAP in school and high myopia, respectively. This pattern indicates that in children with school myopia, differences in pollution exposure have a stronger impact on vision, whereas in severe myopia cases, vision impairment is mainly driven by demographic factors, leaving environmental influences less influential.

Differences were also observed between primary school versus senior school students. As shown in Fig. 5B and C, primary school children on average exhibited better vision (predicted mean UCVA ∼4.75 in our sample) than senior students (mean UCVA ∼4.49). Environmental exposures had a net negative contribution to primary school students’ UCVA (23.8%), while both demographic and habit factors contributed positively. For senior school students, however, environmental factors made a small positive contribution to their UCVA (3.6%), whereas demographic (84.4%) and habit (12.0%) factors had negative contributions.

### Benefits of improving air quality on UCVA

**Fig. 6.**
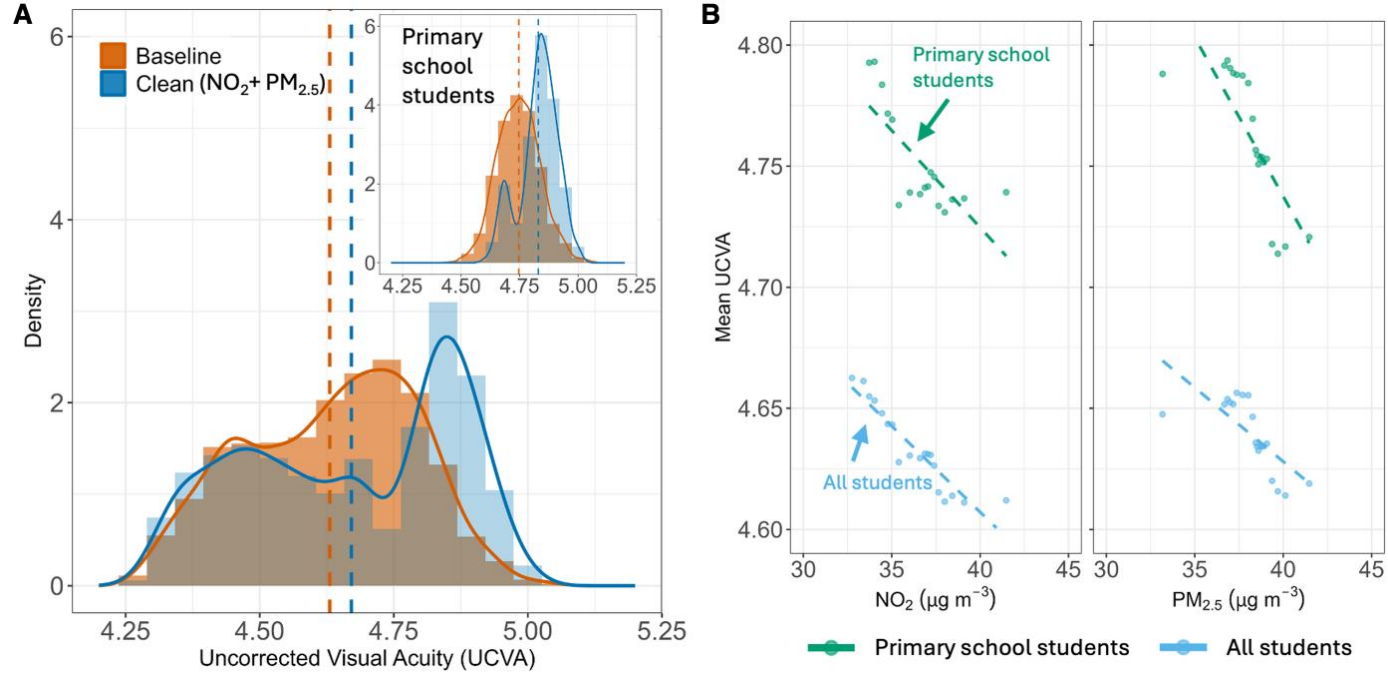
Benefits of improving air quality for UCVA. A) Distribution of UCVA under baseline conditions (Baseline) compared to a scenario in which both annual NO_2_ and PM_2.5_ for the whole population are reduced to the levels currently experienced by the lowest-exposure 20% (Clean). Cleaner air results in improved UCVA, with more pronounced changes for primary school students, as shown in the inset. B) Scatter plots show the relationship between pollutant levels (NO_2_ and PM_2.5_) and mean UCVA. The negative correlation indicates that higher pollutant levels are associated with lower UCVA. Primary school students exhibit greater sensitivity to changes in pollutant levels compared to older students.

We constructed a hypothetical “clean air” cohort (see Methods, below) to quantify the potential vision benefits of clean air actions. We simulated “clean air” scenarios in which ambient annual pollutant concentrations were reduced to the levels currently experienced by the lowest-exposure 20% from ∼35.9 to 31.8 μg m^−3^ for NO_2_ and from ∼38.3 to ∼36.2 μg m^−3^ for PM_2.5_. Then, we compared the predicted UCVA outcomes to the current baseline. Figure 6A shows that the distribution of vision outcomes shifts noticeably toward better acuity under improved air quality for both NO_2_ and PM_2.5_, with more children achieving UCVA in the 4.8–5.0 range from middle of the distribution. The average UCVA of the whole population improved by ∼0.04 units to 4.67 from the current condition (4.63). Removing a single pollutant yields smaller but still meaningful benefits for UCVA as shown in Fig. S5 (i.e. around 0.03- and 0.02-unit improvement under NO_2_-only and PM_2.5_-only scenarios). Nonetheless, the plots also show that the changes in students with high myopia (left tail of the distribution, UCVA ∼4.3–4.5) were less pronounced.

Importantly, younger students would benefit more from cleaner air. The insets of Fig. 6A show that primary school students improve the most in every clean air scenario. Primary students’ mean UCVA is predicted to increase by roughly 0.09 from about 4.74 to 4.83, which is double the improvement observed for the overall student population. Indeed, regression analysis of UCVA against pollutant levels (Fig. 6B) shows that the slope is steeper for primary school students, indicating their higher sensitivity to air quality changes.

## Discussions

### Integrated environmental, genetic, and lifestyle influences on UCVA

Using explainable machine learning model (GBM with SHAP), we identified a combination of environment, genetic, and lifestyle factors as key drivers of UCVA in school-aged children. The mean absolute SHAP rankings (Fig. 2) showed that parental myopia was the top factor affecting children's UCVA (Fig. 2), and it has more significant impacts in severe cases of myopia compared to milder, school-associated forms, consistent with previous studies (4, 15, 16). Gender differences were also observed. Girls exhibited poorer vision relative to boys likely due to their relatively less outdoor activities (17, 18). Additionally, surrounding greenness (NDVI) and lifestyle variables, including nightly sleep duration, homework burden, and frequency of sports activity also showed clear influence on UCVA (Fig. 3B). This underscores that school environment, family history, environmental exposures, and daily habits collectively influence children's visual acuity, rather than any single factor.

Different population groups exhibit varying responses in UCVA to influencing factors and therefore could benefit from different interventions. Children with myopic parents tended to have poorer UCVA (Fig. 3A), reflecting a well-known genetic predisposition (19, 20). They might require more frequent eye examinations and targeted interventions, enabling early detection and management to prevent rapid progression and preserve visual health through timely corrective measures; lifestyle factors had more pronounced effects on middle- and high-school students compared to younger children; the different sensitivities of students to air pollution shown by the steeper slopes in Fig. 6B indicate that air pollution could have different effects on UCVA in different age groups, which will be explored in the next section.

### Differential impact of air pollutants by age group

Our subgroup analysis indicates that the detrimental effect of air pollution on visual acuity varies by age. UCVA of younger children (primary school age) benefits more from air quality improvement (Fig. 5A). This age-dependent disparity is biologically plausible because younger eyes are still developing and therefore more susceptible and vulnerable to pollutant-related damage (21). However, their visual system retains a high degree of plasticity and capability for recovery, potentially allowing greater restoration of visual function when pollution exposure is reduced (22). Additionally, because primary school students typically spend more time outdoors, they are exposed to higher levels of ambient air pollution; accordingly, improvements in air quality may bring greater benefits to this group (23, 24). By contrast, older students engage more in near-work activities (e.g. indoor computer use and intense study schedules), implying a large impact from demographic and educational factors (25, 26). Taken together, the subgroup analyses highlight that the impacts of modifiable risk factors on children's vision are relatively more important in the early stages. Younger children have a higher fraction of their UCVA determined by environment and behavior, suggesting greater potential benefits from protective interventions. In contrast, for older students, vision outcomes are largely governed by unmodifiable factors (e.g. genetics and prior history), meaning interventions like pollution reduction would yield only limited improvements in that group.

### Air quality as a modifiable risk factor for myopia

The focus of this study is the role of ambient air quality (i.e. NO_2_ and PM_2.5_) in childhood visual health. Our results position air pollution alongside (and distinctly from) genetic and behavioral factors in myopia risk (Figs. 2 and 3). Although the magnitude of the vision improvement from clean air is modest in comparison to the influence of genetic and established lifestyle (screen time, reading, etc.), air quality can be improved through policy and infrastructure changes, making it an important modifiable risk factor. From a public health perspective, the finding that poorer air quality is associated with worse UCVA in children adds urgency to efforts to reduce emissions and pollution exposure. Cleaner air may confer dual benefits: directly, by lessening the pollutant-induced ocular stress that can promote axial elongation, and indirectly, by enabling safer outdoor play and greater sunlight exposure for children's eyes. Biologically, there are plausible mechanisms by which chronic exposure to pollutants could influence eye growth.

NO_2_, a significant pollutant arising from emissions of NO_X_ (a summation of nitrogen oxide (NO) and NO_2_) from power stations, motor vehicles, and industrial processes (27), impacts ocular health through direct oxidative stress and inflammation, leading to conditions like conjunctivitis, and indirect reduction of outdoor light exposure, which decreases retinal dopamine release (28, 29). Exposure to PM_2.5_ has been shown to increase ocular surface inflammation, marked by elevated reactive oxygen species and apoptosis of corneal epithelial cells (30). This inflammation disrupts corneal epithelial tight junctions, allowing cytokines like IL-6 and TNF-α to penetrate the cornea and stimulate retinal pigment epithelial cells to secrete more inflammatory cytokines (31). This cascade upregulates matrix metalloproteinase-2 and transforming growth factor-beta, crucial for extracellular matrix remodeling and scleral elongation, leading to abnormal eye elongation and myopia (32, 33).

Therefore, the association observed in our cohort is supported by mechanistic evidence, lending credibility to the idea that reducing children's exposure to NO_2_ and PM_2.5_ should help curb early myopic changes. Our data suggest that improving air quality could form part of a comprehensive strategy to combat the rising prevalence of myopia in urban youth (29, 32).

Because school-age children spend a substantial part of each weekday on or near the school campus (e.g. around 10 h daily), interventions at and around schools can make a meaningful contribution to lowering their cumulative air-pollution dose (Fig. 6). Examples include (i) installing portable or central air-cleaning units inside classrooms as has been trialed in the United Kingdom and several Chinese provinces; (ii) creating or strengthening “clean-air zones” that limit high-emission vehicles on streets adjacent to densely clustered schools; and (iii) scheduling low-traffic “school streets” during arrival and dismissal times. These measures can operate alongside city- or region-wide emission-control policies and may yield additional co-benefits such as quieter learning environments (34), better school performance (35), and improved ocular and overall health (36).

### Limitations

Despite the robust findings, this study has certain limitations. Firstly, the reliance on self-reported data for lifestyle habits, such as sleep duration and homework hours, may introduce reporting bias. Although objective measures, such as wearable devices, could provide more accurate data for some variables (e.g. sleep), capturing others, such as detailed homework activities or lifestyle habits, may be inherently challenging. Future studies may benefit from integrating a combination of self-report and objective monitoring where feasible to balance accuracy and practicality. Secondly, we used ambient air quality as a proxy for individual exposure. This approach may not fully capture indoor exposures, which are challenging to measure. Additionally, studying a single city, where air pollution levels vary less than in regions with larger differences, may limit the generalizability of the findings to other regions with different environmental and socioeconomic conditions. Thirdly, we are not able to precisely align individual exposure data with health outcomes, a common challenge in air pollution epidemiology (37). Future research could focus on longitudinal and intervention studies to further elucidate causal relationships and evaluate the effectiveness of targeted strategies in reducing myopia.

In conclusion, our study highlights the magnitude and variability (i.e. the multimodal distribution) of intrinsic and extrinsic factors in childhood myopia. By using explainable machine learning, we quantified the relative impact of genetic predisposition, environmental exposures, and lifestyle habits on visual acuity. The important association between air pollution and decreased UCVA, particularly among younger students, underscores the potential benefits of environmental interventions as part of comprehensive myopia control strategies. Enhancing air quality around schools, expanding green spaces, and promoting healthy lifestyle practices may help safeguard children's visual health.

## Methods

### Study population and eye examinations

This study included a total of 29,971 participants, recruited from 2021 March 1 to 2023 December 31, in Tianjin, China. Participants were selected from a school-based screening program for myopia among primary, middle, and high school students, namely, Tianjin Child and Adolescent Research of Eye. Written informed consent was obtained from parents or guardians, and the study was conducted in accordance with the Declaration of Helsinki, with approvals from the Institutional Review Board of Tianjin Medical University Eye Hospital. The enrollment of the participants is presented in Fig. S1.

Myopia screening was conducted using a standardized protocol by trained healthcare professionals or school nurses, including visual acuity measurement and refractive examinations. The uncorrected visual acuity (UCVA) of each eye was measured at a distance of 5 m using a standard logarithmic visual acuity E chart. Noncycloplegic autorefraction procedures (Tianle RM-9600, Shanghai, China) involved spherical power, cylindrical power, and axis measurements. Each examination was performed three times for each eye of the students, and the average value was adopted. Further details have been previously reported (38). Myopia was defined as spherical equivalent refraction of ≤−0.50 diopters when UCVA was below 5.0 (39). The spherical equivalent was calculated by summing the sphere power with half of the cylinder power. If either eye of a student was diagnosed with screening myopia, the student was considered myopic in this study. Details of study population and eye examinations were included in Supplementary methods.

### Data collection

#### Demographic and questionnaire variables

Before screening, schoolteachers collected student demographics like age, sex, and educational level. Parents completed questionnaires providing socio-demographic data, educational environment, personal behaviors, sleep habits, and family background, including myopia history and child-specific behaviors. Details of questionnaire variables are included in Supplementary methods.

#### Air pollution

The PM_2.5_ data for the years 2021 to 2022 were obtained from the Big Data Seamless 1-km Ground-level PM_2.5_ Dataset for China, which integrates ground-based measurements, satellite remote sensing products, atmospheric reanalysis, and model simulations. Ground-based measurements of surface PM_2.5_ and various auxiliary variables were incorporated using a space-time extra-trees model, yielding a strong goodness of fit with a cross-validation coefficient of determination (CV-R^2^) of 0.92 and a RMSE of 10.76 μg m^−3^ (40, 41). Similarly, daily NO_2_ data were derived by accounting for spatiotemporal heterogeneity, integrating spatiotemporally weighted information into the extra-trees and deep forest models. This dataset combined surface NO_2_ observations with satellite tropospheric NO_2_ columns from TROPOspheric Monitoring Instrument and Ozone Monitoring Instrument, producing data at a 1-km resolution. The model for NO_2_ prediction achieved a CV-R^2^ of 0.93 and an RMSE of 4.89 μg m^−3^ (42, 43).

#### Green space

The NDVI assessed green space extent around schools, with higher values indicating denser vegetation. Moderate resolution imaging spectroradiometer sensors on Terra and Aqua satellites provided the necessary imagery, processed with radiometric calibration, atmospheric correction, and orthorectification to calculate NDVI = (NIR (near infrared)—RED (red band))/(NIR + RED) (44). NDVI values were computed for buffer zones of 250, 500, and 1,000 m around each school, updated every 16 days and averaged over 2 years from 2021 to 2022. Details of green space data were included in Supplementary methods.

#### Nighttime light

Tianjin's nighttime light data came from the visible infrared imaging radiometer suite day/night band detectors aboard the joint polar satellite system, provided by the Earth Observation Group (45). These data were processed using the ArcGIS platform, undergoing steps such as cropping and projection transformations. The ArcGIS Mosaic Dataset tool stitched the images into a raster dataset, and the raster extraction tool analyzed the nighttime light data. Here, we utilized luminosity data as a proxy for economic status (46). See detailed data collection methods in Supplementary information.

### Model linking explanatory factors to UCVA

We developed a machine learning model to link explanatory factors to UCVA, using H2O.ai's AutoML platform (47). In the AutoML framework, the effect of each predictor on UCVA represents its unique contribution, with the impact measured after accounting for all the other variables in the model. The AutoML seeks the best function $f_{*}(.)$ from an ensemble of trained machine learning models $\hat{\;f}(.)$ (specifically 30 models in this study) based on a predefined criterion:


(1) $$\{\begin{array}{c} y^{i}-f_{*}^{i}(.)\to 0\\ \;f_{*}\;=\;\mathrm{argmi}n_{\hat{f}\in F^{E}x∼D_{\mathrm{train}}(\mathrm{UCVA},\;x_{\mathrm{demog}},x_{\mathrm{habit}},x_{\mathrm{environ}})} \end{array},$$

where $y^{i}$ denotes the observed UCVA of the *i*th individual (student); $D_{\mathrm{train}}$ represents the training dataset; the explanatory variable $x_{\mathrm{demog}}$ represents the demographical information of school students, including school type, parental myopia, sex, and BMI; $x_{\mathrm{habit}}$ are habit-related terms, including sleep duration, homework hour, outdoor hour, rubbing, ballgame, reading distance, green frequency, seat row, transportation, seafood intake, beverage intake, and salt intake; and $x_{\mathrm{environ}}$ represents the environment students are exposed to, including nightlight, NDVI, NO_2_, and PM_2.5_ levels. All integer-coded categorical variables (e.g. sex, school type) were cast as factors before model training, no normalization is applied during model fitting, and the model treats each factor level as an unordered category. Our training and test datasets were generated using a standard 80/20 split, allocating 80% of the data for training and using the remaining 20% cohort data for testing.

We evaluated several regression models including extremely randomized trees (XRT), extreme gradient boosting (XGBoost), and gradient-boosted trees (GBM). We chose RMSE, systematic RMSE (RMSEs), unsystematic RMSE (RMSEu), and index of agreement (48) as the model comparison metrics to examine how each model performs on both the training and test datasets. Table S1 show the hyperparameters used in these machine learning models, alongside their corresponding training and testing performance metrics for the optimally tuned models. As shown in Table S5, the XRT, XGBoost, and GBM are tree-based regression approaches, and our results indicate that GBM models generally outperformed (RMSE = 0.3030) others in terms of predictive accuracy, followed by the XGBoost model (RMSE = 0.3044). Despite XRT model showing a lower RMSE on the training data, GBM model demonstrated improved performance on the test dataset with lower RMSEu, indicating greater reliability compared to other models. Therefore, we selected the GBM model with the best performance for further analysis and interpretation.

## Data analysis

### Feature importance and its relationship with UCVA

We used mean absolute SHapley Additive exPlanations (SHAP) to evaluate the importance of each feature affecting visual acuity. SHAP values, derived from cooperative game theory (49), quantify the contribution of each feature to the difference between the model prediction based on the complete feature set and the mean prediction across all possible feature subsets (48, 50). For our selected model $f_{*}(.)$ and the *i*th school student represented by a feature vector $x^{i}=(x_{\mathrm{demog}}^{i},\;x_{\mathrm{habit}}^{i},\;x_{\mathrm{environ}}^{i})$, the SHAP value for a specific feature *p* is computed as follows:


(2) $$\phi_{p}(\;f_{*},x^{i})=\underset{S\subseteq N\setminus \{p\}}{\sum }\frac{|S|!(|N|-|S|-1)!}{|N|!}\;\;(\;f_{*}(x_{S}^{i}\cup \{x_{p}^{i}\})-f_{*}(x_{S}^{i})).$$

Here, $x_{S}$ denotes the instance *x* with only the features in subset *S* present; *N* is the set of all input features; S⊆N∖{p} represents any subset features excluding feature *p*; $f_{*}(x_{S}^{i})$ is the model prediction using only the features in subset *S*; and $f_{*}(x_{S}^{i}\cup \{x_{p}^{i}\})$ is the model prediction when the feature subset *S* is augmented with *p*. By averaging the absolute SHAP values for feature *p*, we obtained a measure of its global importance, termed $I_{p}$ or mean |SHAP|, as:


(3) $$I_{p}=\frac{1}{m}\overset{m}{\underset{i=1}{\sum }}\;|\phi_{p}(\;f_{*},x^{i})|,$$

where *m* is the total number of instances in the dataset D. The concept of “mean |SHAP|” focuses on the magnitude of feature attribution, offering a more accurate and detailed measure of feature importance compared to traditional feature importance, which is based on the decrease in model performance when feature values are randomly shuffled. The SHAP summary was provided to illustrate both the magnitude and direction of the feature effects for each feature across all predictions. To further indicate the marginal relationship between each feature and UCVA, we employed partial dependence plot, which gives a visual representation of the predicted outcomes over a range of values or factors for the feature of interest while averaging out the influence of all other features.

### Local explanation for subgroup analysis

We performed a subgroup analysis using the “mean |SHAP|” and local SHAP explanations. Initially, SHAP values were calculated for individual instances, such as assessing how different features contributed to the predictions for a single student in this study. We then extended this local explanation approach to specified groups. Specifically, we define the average SHAP value for a subgroup $D_{K}$ as follows:


(4) $$\bar{\;f_{*}}(D_{K})=\frac{1}{k}\underset{i\in D_{K}}{\sum }\;\left(\phi_{0}+\overset{n}{\underset{\;p=1}{\sum }}\phi_{p}(\;f_{*},x^{i})\right)$$

$\bar{\;f_{*}}(D_{K})$ represents the average SHAP value for the subgroup $D_{K}$; *k* is the number of individuals in $D_{K}$; $\phi_{0}$ is the mean prediction over the entire dataset D; and *n* is the total number of features.

We divided our dataset into subgroups based on specific characteristics, including the degree of myopia (“high myopia” versus “school myopia”) and school type (primary school students versus high-school students). The modified local SHAP explanations enable us to evaluate the average impact of each feature, or a group of features, on a subgroup rather than just individual predictions, and it helps us identify which features are more influential for different groups of students.

### Air quality scenarios

We conducted a counterfactual analysis to determine the impact of air quality on UCVA using the following formula:


(5) $${\bar{y}}_{D_{R}}^{i}=\frac{1}{t}\overset{t}{\underset{\;j=1}{\sum }}\;f_{*}(x_{\mathrm{demog}}^{i},x_{\mathrm{habit}}^{i},x_{\mathrm{environ}\setminus \{p\}}^{i},x_{p}^{\;j\in D_{R}}),$$

where *p* represents the concentrations of PM_2.5_ and NO_2_, which are the key pollutants of interest; ${\bar{y}}_{D_{R}}^{i}$ is the averaged value of UCVA for the *i*th individual, obtained by resampling $x_{p}$ from the subgroup dataset $D_{R}$; and *t* is the total number of resampling iterations, set at 500 for this study to balance computational feasibility with statistical robustness. Each iteration *j* involves randomly replacing $x_{p}$ for individual *i* with $x_{p}$ from another member of the subgroup dataset $D_{R}$.

Using original model predictions as a baseline, we constructed a “clean air” scenario by selecting the subgroup $D_{R}$ to include the lowest 20% of observed pollutant concentrations. This scenario evaluates the potential benefits of improved air quality on UCVA across different demographic cohorts, including all student populations and a subset comprising only primary school students. We assessed three interventions: reducing PM_2.5_ levels, reducing NO_2_ levels, and a combined reduction of both pollutants. Additionally, we segmented the pollution data into 20 finer subgroups, enabling a thorough analysis of trends and potential correlations between different levels of air pollution exposure and average UCVA outcomes. This segmentation helps to understand the potential negative impacts associated with worsening air quality.

### Sensitivity analyses

We conducted several analyses to assess the robustness of our main findings. Firstly, we switched the response variable to a binary myopia diagnosis while retaining the same predictor variables and model configurations. This adaptation resulted in a good performance, with an area under the curve of 0.76 as showed in the Supplementary information (Fig. S4). Secondly, we explored the stability of our results by implementing alternative modeling approaches. We compared the performance of an extremely randomized trees (XRT) model and an XGBoost model against the model used in our study. Both models offer consistent results, indicating that our main discussions are reliable across different modeling approaches and model choices (Figs. S6 and S7). Additionally, we conducted an analysis using a traditional multivariate logistic regression model adjusted for all influential variables in this study. Although the regression could not represent the higher-order interactions that the SHAP analysis revealed, it recaptured the same directions of effect with odd ratios (Table S6). These comparisons show that AutoML provides better prediction accuracy and richer insight into the impact of each factor on UCVA, while the consistency across methods underscores the robustness of our central finding.

All analyses were conducted on the BlueBEAR HPC service (https://www.birmingham.ac.uk/research/arc/bear/bluebear) at University of Birmingham. R software (version 4.3.3) was used for all model operations.

## Supplementary Material

pgaf279_Supplementary_Data

## Data Availability

Air quality data are sourced from https://zenodo.org/communities/chap/records?q=&l=list&p=1&s=10&sort=newest (CHAP) and https://weijing-rs.github.io/product.html. NDVI data are from https://ladsweb.modaps.eosdis.nasa.gov/search/order/1. Nightlight data are from https://eogdata.mines.edu/products/vnl/. All data utilized in this study are openly accessible on GitHub (https://github.com/clnair-ascm/aq2myopia).
