# Supplementary material for: Benefits of clean air for school children's vision health
Source: PNAS Nexus. 2025 Sep 23;4(9):pgaf279. doi: 10.1093/pnasnexus/pgaf279 (PMC12455593; doi:10.1093/pnasnexus/pgaf279)
Supplement: pgaf279_Supplementary_Data [file pgaf279_supplementary_data.docx]

**Supporting Information for**

Benefits of Clean Air for School Children’s Vision Health

Xi Chen ^a1^, Yuqing Dai ^b1^, Ruihua Wei ^c^, Bei Du ^c^, Congchao Lu ^a^, A. Robert MacKenzie ^b^, Nai-jun Tang ^a*^, Zongbo Shi ^b*^, Hua Yan ^d*^.

^a^ Department of Occupational and Environmental Health, School of Public Health, Tianjin Medical University, Tianjin, China.

^b^ School of Geography, Earth and Environmental Science, University of Birmingham, Birmingham, B15 2TT, U.K.

^c^ Tianjin Key Laboratory of Retinal Functions and Diseases, Tianjin Branch of National Clinical Research Center for Ocular Disease, Eye Institute and School of Optometry, Tianjin Medical University Eye Hospital, Tianjin, China.

^d^ Department of Ophthalmology, Tianjin Medical University General Hospital, International Joint Laboratory of Ocular Diseases (Ministry of Education), Tianjin Key Laboratory of Ocular Trauma, Tianjin Institute of Eye Health and Eye Diseases, China-UK "Belt and Road" Ophthalmology Joint Laboratory, Laboratory of Molecular Ophthalmology, Tianjin Medical University, Tianjin, China.

*** Corresponding authors:**

Hua Yan, Zongbo Shi, Nai-jun Tang

**Email:** [zyyyanhua@tmu.edu.cn](mailto:zyyyanhua@tmu.edu.cn), [Z.Shi@bham.ac.uk](mailto:Z.Shi@bham.ac.uk), [tangnaijun@tmu.edu.cn](mailto:tangnaijun@tmu.edu.cn)

**^1^ X.C. and Y.D. contributed equally to this work.**

**This PDF file includes:**

Supporting Information Text

Figures S1 to S6

Tables S1 to S6

SI References

Supporting Information Text

**S1. Demographic and Questionnaire Variables.** Demographic information such as age, sex, and educational level was collected by schoolteachers before the screening. Socio-demographic data, students' educational environment, personal behavioral patterns, sleep habits, and family background were collected through questionnaires completed by parents. These questionnaires also included items on the family history of myopia and specific children’s behaviors.

The schools were categorized as urban, suburban, or rural based on their geographical location. Variables included physical activity, daily near work duration, length of sleep, and diet habits of students. If one or both parents had myopia, the student was considered to have a family susceptibility. Additionally, the frequency of students' weekly consumption of desserts, seafood, and drinks was divided into two categories.

| Variables | Questions | Options |
| --- | --- | --- |
| Parental myopia | Is the child’s mother myopic? | Yes |
|  |  | No |
|  | Is the child’s father myopic? | Yes |
|  |  | No |
| Sleep duration | In the last month, what time do you usually go to bed at night? | Around ___: ___ at night. |
|  | In the last month, what time do you usually get up? | Around ___: ___. |
| Homework hour | How long does it take you on average to do your homework each day？ | ____ hours/day. |
| Rubbing | Do you often rub your eyes? | Never |
|  |  | Occasionally |
|  |  | Often |
|  |  | Always |
| Ballgame | What ballgame do you usually paly? | Table tennis |
|  |  | Badminton |
|  |  | Volleyball |
|  |  | Football |
|  |  | Basketball |
|  |  | None of the above |
| Reading distance | Do you read at a distance greater than one foot (33cm) in your daily life? | Yes |
|  |  | No |
| Seat row | Which row do you sit in the classroom? | 1~3 rows |
|  |  | 4~6 rows |
|  |  | 7~10 rows |
| Green frequency | How often does the child visit a park, forest, or other green spaces? | Almost every day |
|  |  | About once a month |
|  |  | 2-3 times per month |
|  |  | Once every few months |
|  |  | Never |
| Seafood intake | What is the frequency of your child's intake of fish, shrimp, crab, shellfish, or other aquatic products? | Hardly ever |
|  |  | Less than 1 time/week |
|  |  | 2-3 times/week |
|  |  | 4-6 times/week |
|  |  | 1 time/day |
|  |  | 2 or more times/day |
| Transportation | What kind of transportation do you usually choose to go to school? | Walking |
|  |  | Bicycle |
|  |  | Subway |
|  |  | Bus |
|  |  | Private car |
|  |  | Living in school |
| Beverage intake | What is the frequency of your child's intake of carbonated beverages (sodas, colas, etc.) and milk tea? | Hardly ever |
|  |  | Less than 1 time/week |
|  |  | 2-3 times/week |
|  |  | 4-6 times/week |
|  |  | 1 time/day |
|  |  | 2 or more times/day |
| Salt intake | What is the salt content of your child's diet? | Less than 4g/day (lighter taste) |
|  |  | 4-6g/day (normal taste) |
|  |  | Above 6 g/day (salty taste) |
| Outdoor hour | What is the total number of hours you spend outdoors (including any activity, not limited to sports) each day? | <1h |
|  |  | 1-2h |
|  |  | 2-3h |
|  |  | ≥3h |

**S2. Study Population.** The TCARE (Tianjin Child and Adolescent Research of Eye) study is a large-scale, population-based dynamic cohort study conducted in Tianjin, China. The project is based on a school-based screening program for myopia among primary and secondary school students. It has been designed as a long-term project with the aim of annual follow-up. The TCARE study aims to identify the determinants that contribute to the development of myopia and to better understand its progression in school-aged children and adolescents who are at risk of developing myopia.

The study included participants who were students enrolled in primary schools, middle schools, and high schools across sixteen districts in Tianjin from March 1, 2021, to December 31, 2023. Students who have previously undergone cataract surgery, laser refractive surgery, or low-dose atropine treatment were excluded from this study. Written informed consent was obtained from parents or guardians participating in the study. This study was performed in accordance with the Declaration of Helsinki, with approvals from the Institutional Review Board of Tianjin Medical University Eye Hospital [(2020) 404]. Written informed consent was obtained from each participating individual’s guardian.

**S3. Procedures.** From March 1, 2021, to December 31, 2023, the study enrolled 2,087,074 ostensibly healthy school-aged children from 1,589 schools across China. During this period, 90,111 students were excluded from the analysis due to graduation in 2021. An additional 272,116 new students joined, yielding a total cohort of 1,258,668 in 2022. The enrollment and screening procedures are presented in Figure S1. Myopia screening was conducted for school students using a standardized protocol by trained healthcare professionals or school nurses, including visual acuity measurement and refractive examinations. The uncorrected visual acuity (UCVA) of each eye was measured at a distance of 5 m using a standard logarithmic visual acuity E chart. Non-cycloplegic autorefraction procedures (Tianle RM-9600, Shanghai, China) involved spherical power, cylindrical power, and axis measurements. Each examination was performed three times for each eye of the students, and the average value was adopted. Further details have been previously reported (1).

**S4. Outcomes.** Myopia was defined as spherical equivalent refraction of ≤ -0.50 diopters when UCVA was below 5.0 (2). The spherical equivalent was calculated by summing the sphere power with half of the cylinder power. If either eye of a student was diagnosed with screening myopia, the student was considered myopic in this study. The primary outcome was the prevalence of myopia, defined as the proportion of students who were myopic during the final visit in the year 2022. The secondary outcome indicated the progression of myopia in this cohort during the one-year follow-up, including the incidence of myopia and the decline in both visual acuity levels and spherical equivalent refraction (SER). The incidence of myopia was defined as the proportion of students who did not have myopia at baseline but developed it at the final visit. The decline in visual acuity levels was defined as the change in UCVA, and the decline in SER was defined as the change in SER during the follow-up period.

**S5. Green Space.** The Normalized Difference Vegetation Index (NDVI) was utilized to assess the extent of green spaces within the school using remotely sensed data. NDVI values, which range from -1 to 1, reflect the density of vegetation, with higher values indicating more extensive vegetation cover. Remote sensing images captured in the visible red and near-infrared bands were obtained from the Moderate-Resolution Imaging Spectroradiometer (MODIS) sensor on board the Terra and Aqua polar-orbiting satellites, which are part of the U.S. Earth Observing System (EOS). These images were calculated after radiometric calibration, atmospheric correction, and orthorectification (PRC parameters) according to the following equations: NDVI = (NIR-RED)/(NIR + RED), where NIR represents the near-infrared band and RED denotes the visible red band (4).

School addresses were obtained from publicly available school addresses in the Tianjin education system. The NDVI was then calculated for concentric buffer zones around each school at distances of 250 meters, 500 meters, and 1000 meters, with assessments repeated every 16 days. The choice of these distances was based on typical walking distances in urban China, where a 1-kilometer radius generally represents approximately a 15-minute walk. An average NDVI value was then calculated for each buffer zone over a three-year period from 2020 to 2022.

**S6. Nighttime Light.** The acquisition of Tianjin night lighting data is realized through the ArcGIS platform. The data were obtained from the Earth Observation Group (EOG) of the Colorado School of Mines. The data were obtained through remote sensing satellite observations, and the visible-band images were remotely interpreted and image-processed. The Visible and Infrared Imaging Suite (VIIRS) Day/Night Band (DNB) on board the latest generation of Earth observation satellites, the Joint Polar-orbiting Satellite System (JPSS), measures nighttime visible and near-infrared light on a daily basis (5). First, the Colorado School of Mines' EOG website downloads nighttime illumination image data for a specified time period. After downloading the data, data preprocessing is required. Since the data were already atmospherically corrected, only image processing such as cropping and projection transformations were required to fit the study area and analysis requirements. Then, the preprocessed black dolomite nighttime light images are stitched together to generate a complete nighttime light image covering Tianjin and the surrounding areas. The Mosaic Dataset tool in ArcGIS is used to stitch multiple images into a raster dataset. Finally, the raster extraction tool in the ArcGIS platform is used to extract the night lighting data of Tianjin from the stitched night lighting images and store and analyze them in raster data format.

**S7. Statistical Analysis using a Logistic Regression Model.**

Participants data were presented as continuous or categorical variables. Kolmogorov-Smirnov test was used to assess whether the data followed a normal distribution:

$D=max\{{max}_{i} [i / n - F(X(i))],{max}_{i} [F(X(i)) - (i - 1) / n)]\}$ (S1)

$F(x)$ is the probability distribution function of the normal distribution.

The continuous variables involved in this study are all non-normally distributed, the Mann-Whitney U test was used, and results were presented as median (interquartile range):

$Z=\frac{W-\frac{n(n+m+1)}{2}}{\sqrt{\frac{nm(n+m+1)}{12}}}$ (S2)

W is the Mann-Whitney test statistic, *n* and *m* are sample sizes. Categorical data were presented as numbers and frequencies, and either the Chi-square test or Fisher's exact test was used for comparisons:

$\chi^{2}=\sum_{i=1}^{r} \sum_{j=1}^{c} \frac{(O_{ij}-E_{ij})^{2}}{E_{ij}}$ (S3)

$O_{ij}$ is the measured frequency of cell (i, j) and $E_{ij}$ is the expected frequency of cell (i, j). When exploring the effect of factors on myopia/non-myopia or different levels of myopia, logistic regression was used to calculate ORs and their 95% CIs：

$P=\frac{e^{\beta_{0}+\beta_{1}x_{1+\beta_{1}x_{1}+\cdot\cdot\cdot+\beta_{k}x_{k}}}}{1+e^{\beta_{0}+\beta_{1}x_{1+\beta_{1}x_{1}+\cdot\cdot\cdot+\beta_{k}x_{k}}}}$ (S4)

${OR}_{j}=e^{\beta_{j}}$ (S5)

All statistical tests were two-sided, with P-values <0.05 indicating statistical significance.

**Supplementary Results**


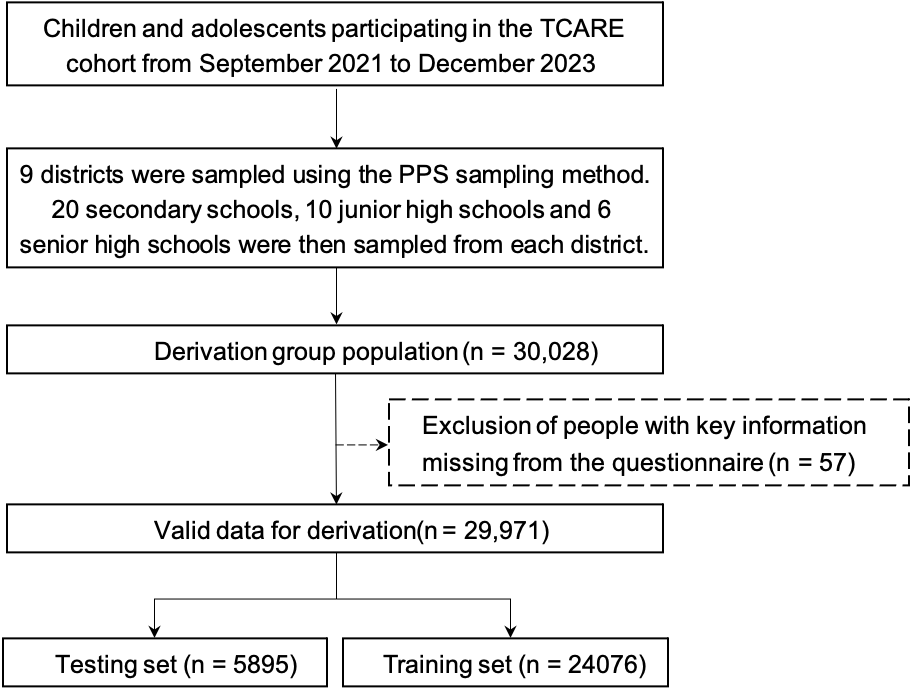


Fig. S1. Flowchart of Participant Enrollment and Study Design. This figure provides a visual representation of the participant enrollment process and the overall study design. It outlines the inclusion criteria, the total number of participants, and the division of the cohort into training and testing sets for the machine learning models.


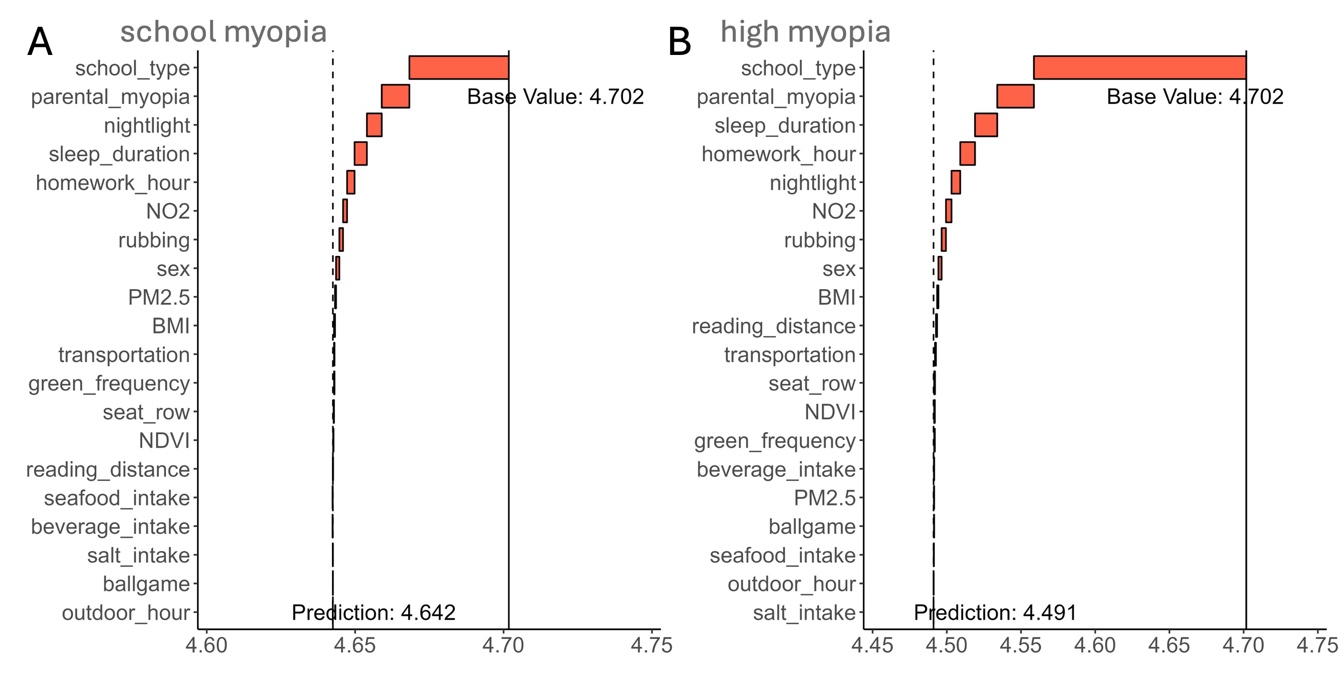


Fig. S2. Impact of Predictive Features on UCVA in Different Myopia Types. Waterfall chart illustrates the step-by-step contribution of each feature to the prediction of UCVA for students with (A) school myopia and (B) high myopia. Blue bars indicate positive contributions and orange bars indicate negative contributions.


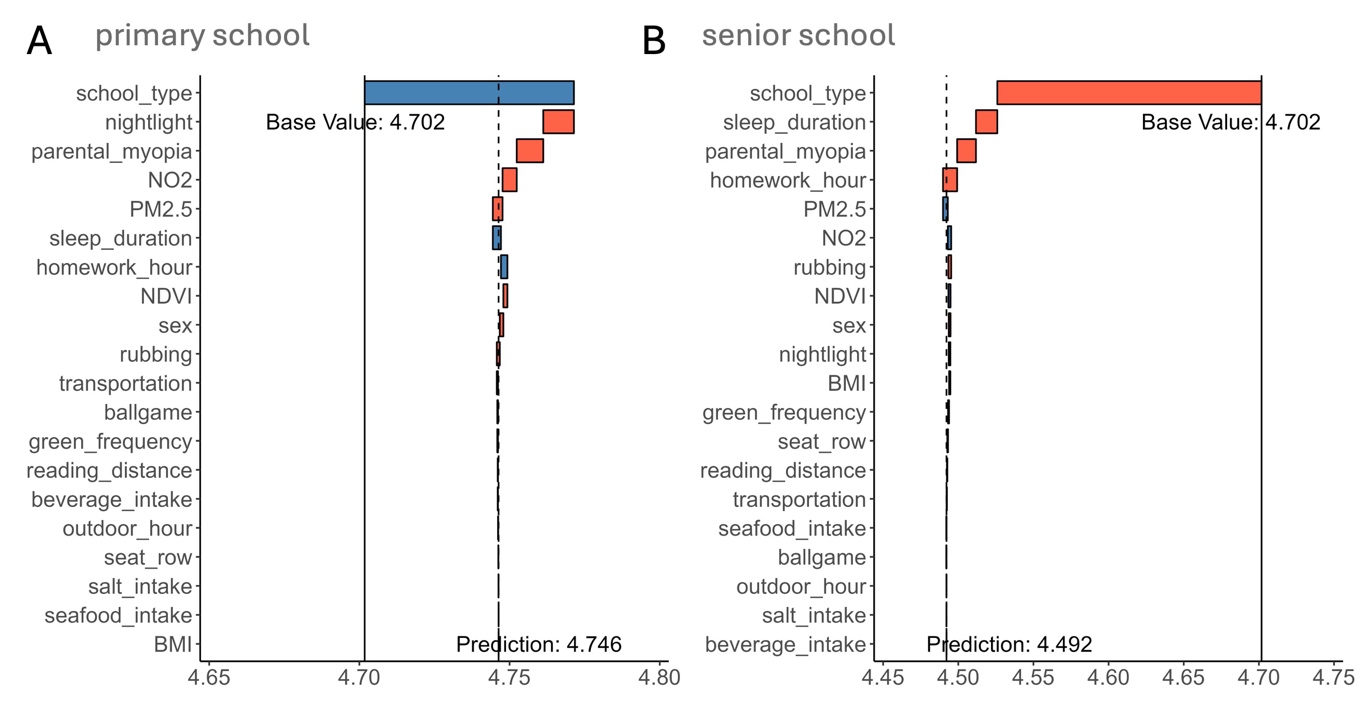


Fig. S3. Impact of Predictive Features on UCVA in Different School Types. The left panels in both Waterfall chart illustrates the step-by-step contribution of each feature to the prediction of UCVA for students with (A) primary school students and (B) middle and high school students. Blue bars indicate positive contributions and orange bars indicate negative contributions.


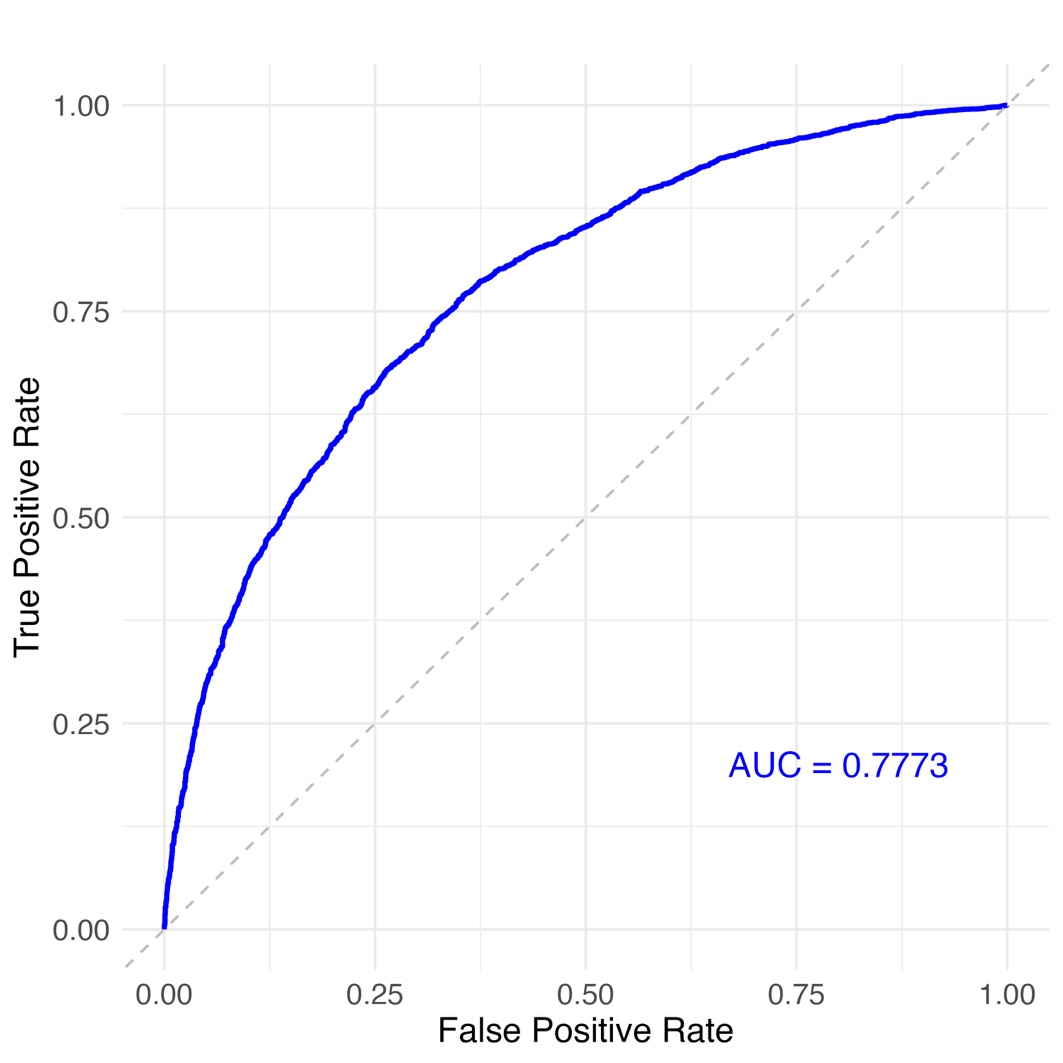


Fig. S4. ROC Curve for Myopia Diagnosis. The receiver operating characteristic (ROC) curve in this figure illustrates the diagnostic performance of the machine learning model when the response variable is binary myopia diagnosis. The area under the curve (AUC) is used as a measure of the model's ability to distinguish between myopia and non-myopia cases.

**
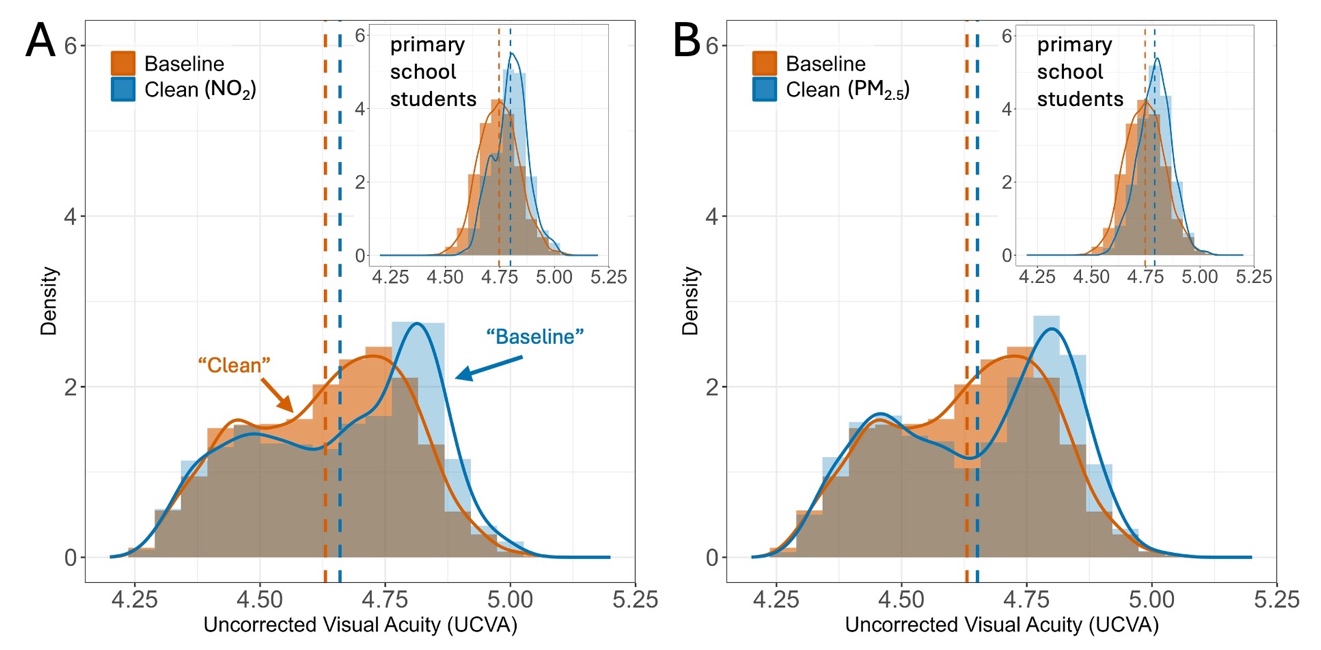
**

**Fig. S5. Benefits of Improving Air Quality for UCVA. (A)** Distribution of UCVA under baseline conditions (orange) compared to a scenario in which annual NO_2_ for the whole population are reduced to the levels currently experienced by the lowest-exposure 20% (blue). Cleaner air results in improved UCVA, with more pronounced changes for primary school students, as shown in the inset. **(B)** Distribution of UCVA under baseline conditions (orange) compared to a scenario in which annual PM_2.5_ for the whole population are reduced to the levels currently experienced by the lowest-exposure 20% (blue).


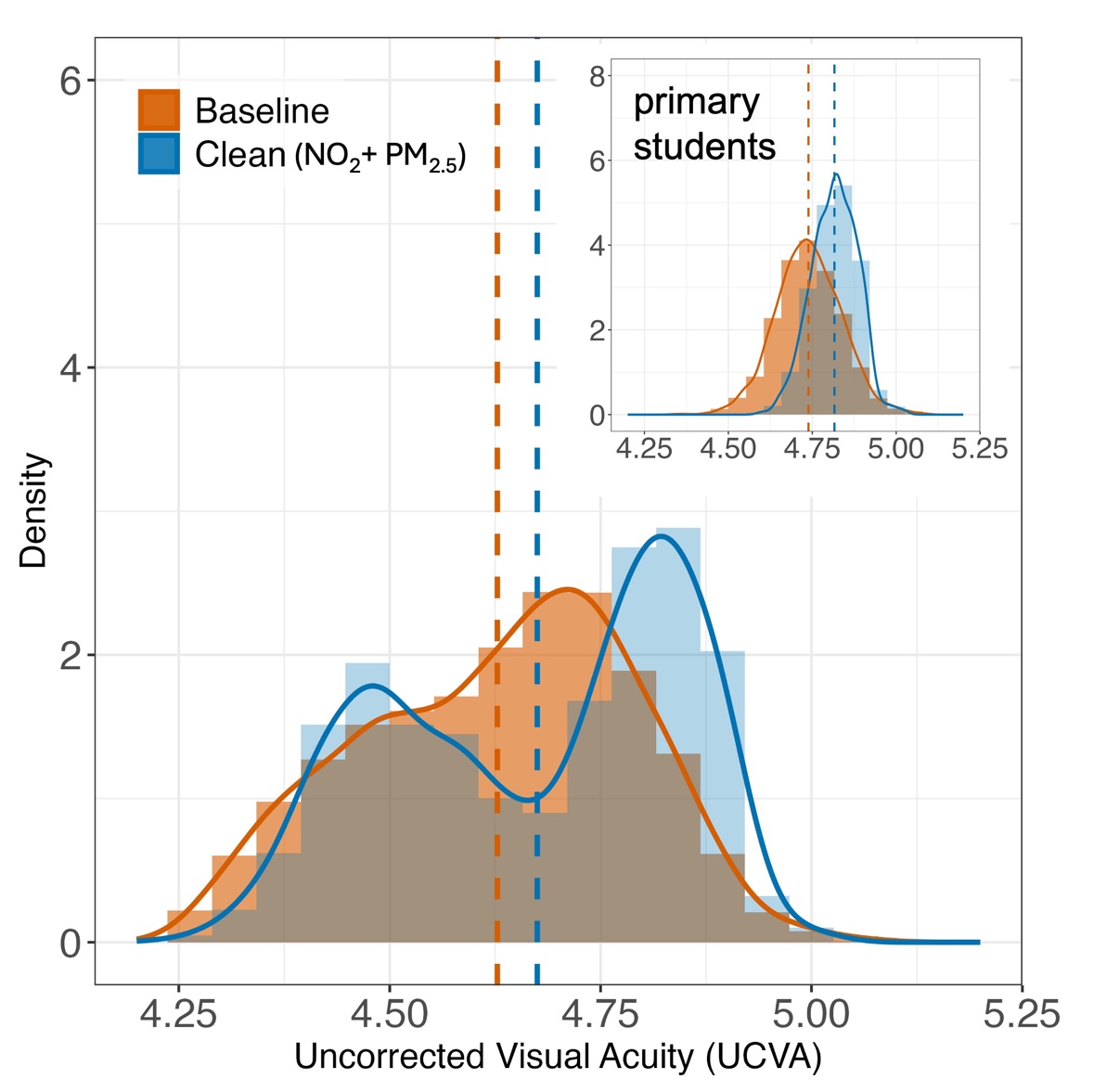


**Fig. S6.** **Benefits of Improving Air Quality (NO_2_ and PM_2.5_) for UCVA using the Extremely Randomized Trees (XRT).**


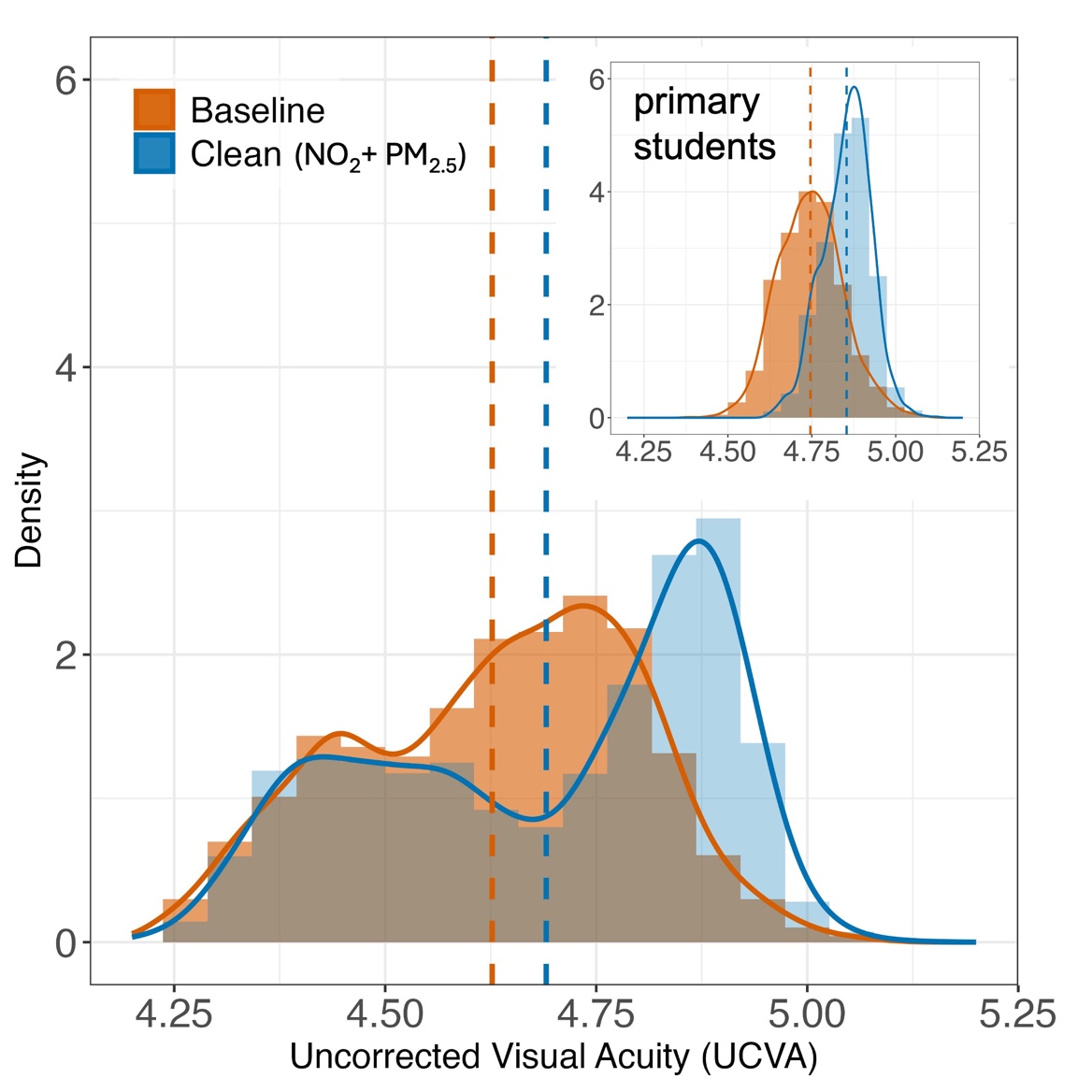


Fig. S7. Benefits of Improving Air Quality (NO_2_ and PM_2.5_) for UCVA using the Extreme Gradient Boosting (XGBoost).

Table S1. All models developed for UCVA predictions from Automated Machine Learning (AutoML) framework.

Table S2. Comparison of demographic and clinical characteristics and outcomes between training and testing set.

| **Variables** | **Test（n=5,895）** | **Training（n=24,076）** | ***P* value** |
| --- | --- | --- | --- |
| UCVA, P_50_ (P_25_, P_75_) | 4.8 (4.4, 5.0) | 4.8 (4.4, 5.0) | 0.057 |
| Sex, n (%) |  |  | 0.333 |
| Female | 2866 (48.6) | 11536 (47.9) |  |
| Male | 3029 (51.4) | 12540 (52.1) |  |
| Parental myopia, n (%) | | | 0.851 |
| Neither | 2087 (35.4) | 8555 (35.5) |  |
| Either | 3808 (64.6) | 15521 (64.5) |  |
| BMI ^a^, P_50_ (P_25_, P_75_) | 17.6 (15.2, 20.3) | 17.6 (15.3, 20.3) | 0.154 |
| Level of education, n (%) | | | 0.916 |
| Primary school | 4126 (70.0) | 16876 (70.1) |  |
| Middle school | 1255 (21.3) | 5074 (21.1) |  |
| High school | 514 (8.7) | 2126 (8.8) |  |
| Mode of commuting to school, n (%) | | | 0.175 |
| Non-motorized transport | 3321 (56.3) | 13878 (57.6) |  |
| Private car | 2108 (35.8) | 8254 (34.3) |  |
| Public transport | 419 (7.1) | 1728 (7.2) |  |
| Residential | 47 (0.8) | 216 (0.9) |  |
| Seat rows, n (%) | | | 0.488 |
| 1-3 rows | 2565 (43.5) | 10533 (43.7) |  |
| 4-6 rows | 2557 (43.4) | 10525 (43.7) |  |
| 7-10 rows | 773 (13.1) | 3018 (12.5) |  |
| Time of homework ^b^, P_50_ (P_25_, P_75_) | 1.00 (1.00, 2.00) | 1.00 (1.00, 2.00) | 0.523 |
| Time of outdoor activities ^c^, n (%) | | | 0.014 |
| ＜1 hour | 1536 (26.1) | 6458 (26.8) |  |
| 1-2 hours | 3268 (55.4) | 12883 (53.5) |  |
| 2-3 hours | 809 (13.7) | 3578 (14.9) |  |
| Reading distance, n (%) |  |  | 0.773 |
| Less than a foot | 1213 (20.6) | 4995 (20.7) |  |
| Greater than a foot | 4682 (79.4) | 19081 (79.3) |  |
| Rubbing eyes, n (%) | | | 0.596 |
| Never | 823 (14.0) | 3449 (14.3) |  |
| Sometimes | 4578 (77.7) | 18649 (77.5) |  |
| Frequent | 456 (7.7) | 1794 (7.5) |  |
| Always | 38 (0.6) | 184 (0.8) |  |
| Ball games, n (%) | | | 0.403 |
| Never | 1686 (28.6) | 6884 (28.6) |  |
| 1 kind | 2714 (46.0) | 11110 (46.1) |  |
| 2 kinds | 1120 (19.0) | 4399 (18.3) |  |
| 3 kinds | 307 (5.2) | 1339 (5.6) |  |
| 4 kinds | 54 (0.9) | 277 (1.2) |  |
| 5 kinds | 14 (0.2) | 67 (0.3) |  |
| Sleep duration ^d^, P_50_ (P_25_, P_75_) | 9.00 (8.50, 9.67) | 9.00 (8.50, 9.67) | 0.591 |
| Frequency of going to green spaces, n (%) | | | 0.231 |
| Never | 248 (4.2) | 1061 (4.4) |  |
| Once every few month | 1151 (19.5) | 4949 (20.6) |  |
| More than 1 time per month | 3445 (58.4) | 13758 (57.1) |  |
| Almost every day | 1051 (17.8) | 4308 (17.9) |  |
| Frequency of soft drinks, n (%) | | | 0.671 |
| Hardly ever | 2215 (37.6) | 8921 (37.1) |  |
| Less than 1 time / week | 2188 (37.1) | 8846 (36.7) |  |
| 2-3 times / week | 1170 (19.8) | 5019 (20.8) |  |
| 4-6 times / week | 188 (3.2) | 733 (3.0) |  |
| 1 time / day | 89 (1.5) | 373 (1.5) |  |
| 2 or more times / day | 45 (0.8) | 184 (0.8) |  |
| Daily dietary salt intake, n (%) | | | 0.644 |
| ＜4 g | 950 (16.1) | 3774 (15.7) |  |
| 4-6 g | 4635 (78.6) | 18995 (78.9) |  |
| ＞6 g | 310 (5.3) | 1307 (5.4) |  |
| Frequency of seafood intake, n (%) | | | 0.837 |
| Hardly ever | 698 (11.8) | 2914 (12.1) |  |
| Less than 1 time / week | 1765 (29.9) | 7158 (29.7) |  |
| 2-3 times / week | 2807 (47.6) | 11556 (48.0) |  |
| 4-6 times / week | 451 (7.7) | 1781 (7.4) |  |
| Once a day | 117 (2.0) | 496 (1.9) |  |
| More than 2 times a day | 57 (1.0) | 198 (0.8) |  |
| Nightlight, P_50_ (P_25_, P_75_) | 52.63 (13.92, 83.44) | 52.63 (13.92, 83.44) | 0.386 |
| NDVI ^e^, P_50_ (P_25_, P_75_) | 0.24 (0.21, 0.29) | 0.24 (0.21, 0.28) | 0.478 |
| PM_2.5_, P_50_ (P_25_, P_75_) | 38.50 (37.25, 38.95) | 38.50 (37.25, 38.95) | 0.496 |
| NO_2,_ P_50_ (P_25_, P_75_) | 36.40 (34.20, 37.50) | 36.45 (34.20, 37.50) | 0.543 |

UCVA, uncorrected visual acuity; BMI, body mass index; NDVI, normalized difference vegetation index; PM_2.5_, the particulate matter smaller than 2.5 micrometers; NO_2_, nitrogen dioxide.

a. BMI was missing in 238 cases in the testing set and 892 cases in the training set.

b. Homework hours was missing in 238 cases in the testing set and 932 cases in the training set.

c. Outdoor hours was missing in 282 cases in the testing set and 1157 cases in the training set.

d. Sleep duration was missing in 189 cases in the testing set and 781 cases in the training set.

e. NDVI was missing in 8 cases in the testing set and 38 cases in the training set.

Table S3. Comparison of demographic and clinical characteristics between primary school and other school types

| **Factors** | **Total** | **Primary school** | **Other school types** | **OR (95%CI)** |
| --- | --- | --- | --- | --- |
|  | **(N=29,971)** | **(n=21,002)** | **(n=8,969)** |  |
| Sex, n (%) |  |  |  |  |
| Female | 14,402 (48.1) | 9,977 (47.5) | 4,425 (49.3) | Reference |
| Male | 15,569 (51.9) | 11,025 (52.5) | 4,544 (50.7) | 0.788 (0.745, 0.833) |
| Parental myopia, n (%) | | | |  |
| Neither | 10,642 (35.5) | 6,823 (32.5) | 3,819 (42.6) | Reference |
| Either | 19,329 (64.5) | 14,179 (67.5) | 5,150 (57.4) | 1.788 (1.685, 1.897) |
| BMI ^a^, IQR | 17.6 (15.2, 20.3) | 17.9 (15.2, 20.3) | 18.1 (15.6, 20.4) | 1.035 (1.027, 1.043) |
| Mode of commuting to school, n (%) | | | |  |
| Non-motorized transport | 17,199 (57.4) | 13,013 (62.0) | 4,186 (46.7) | Reference |
| Private car | 10,362 (34.6) | 7,058 (33.6) | 3,304 (36.8) | 0.919 (0.868, 0.974) |
| Public transport | 2,147 (7.2) | 919 (4.4) | 1,228 (13.7) | 1.032 (0.917, 1.162) |
| Residential | 263 (0.9) | 12 (0.1) | 251 (2.8) | 1.895 (1.095, 3.279) |
| Seat rows, n (%) |  |  |  |  |
| 1-3 rows | 13,098 (43.7) | 9,132 (43.5) | 3,966 (44.2) | Reference |
| 4-6 rows | 13,082 (43.6) | 9,181 (43.7) | 3,901 (43.5) | 0.923 (0.872, 0.978) |
| 7-10 rows | 3,791 (12.6) | 2,689 (12.8) | 1,102 (12.3) | 0.863 (0.792, 0.941) |
| Time of homework ^b^, IQR | 1.0 (1.0, 2.0) | 1.0 (1.0, 1.5) | 2.0 (1.0, 2.0) | 1.118 (1.073, 1.164) |
| Time of outdoor activities ^c^, n (%) | | | |  |
| ＜1 hour | 7,994 (26.7) | 4,610 (22.0) | 3,384 (37.7) | Reference |
| 1-2 hours | 16,151 (53.9) | 11,761 (56.0) | 4,390 (48.9) | 0.956 (0.895, 1.021) |
| 2-3 hours | 4,387 (14.6) | 3,568 (17.0) | 819 (9.1) | 0.921 (0.843, 1.006) |
| Reading distance, n (%) | | | |  |
| Less than a foot | 6,208 (20.7) | 4,345 (20.7) | 1,863 (20.8) | Reference |
| Greater than a foot | 23,763 (79.3) | 16,657(79.3) | 7,106 (79.2) | 0.885 (0.826, 0.948) |
| Rubbing eyes, n (%) | | | |  |
| Never | 4,272 (14.3) | 3,332 (15.9) | 940 (10.5) | Reference |
| Sometimes | 23,227 (77.5) | 16,155 (76.9) | 7,072 (78.8) | 1.350 (1.248, 1.460) |
| Frequent | 2,250 (7.5) | 1,393 (6.6) | 857 (9.6) | 1.762 (1.551, 2.002) |
| Always | 222 (0.7) | 122 (0.6) | 100 (1.1) | 1.290 (0.896, 1.858) |
| Ball games, n (%) | | | |  |
| Never | 8,570 (28.6) | 5.879 (28.0) | 2,691 (30.0) | Reference |
| 1 kind | 13,824 (46.1) | 9,658 (46.0) | 4,166 (46.4) | 0.981 (0.919, 1.048) |
| 2 kinds | 5,519 (18.4) | 3,976 (17.6) | 1,543 (17.2) | 0.960 (0.884, 1.043) |
| 3 kinds | 1,646 (5.5) | 1,210 (5.8) | 436 (4.9) | 1.041 (0.915, 1.185) |
| 4 kinds | 331 (1.1) | 238 (1.1) | 93 (1.0) | 1.048 (0.799, 1.375) |
| 5 kinds | 81 (0.3) | 41 (0.2) | 40 (0.4) | 1.044 (0.587, 1.858) |
| Sleep duration ^d^, IQR | 8.50 (9.00, 9.67) | 9.50 (9.00, 9.92) | 8.33 (7.92, 9.00) | 0.928 (0.889, 0.968) |
| Frequency of going to green spaces, n (%) | | | |  |
| Never | 1,309 (4.4) | 558 (2.7) | 751 (8.4) | Reference |
| Once every few month | 6,100 (20.4) | 3,653 (17.4) | 2,447 (27.3) | 1.069 (0.909, 1.256) |
| More than 1 time per month | 17,203 (57.4) | 12,484 (59.4) | 4,719 (52.6) | 0.947 (0.810, 1.106) |
| Almost every day | 5,359 (17.9) | 4,307 (20.5) | 1,052 (11.7) | 0.906 (0.768, 1.069) |
| Frequency of soft drinks, n (%) | | | |  |
| Hardly ever | 11,136 (37.2) | 8,649 (41.2) | 2,487 (27.7) | Reference |
| Less than 1 time / week | 11,034 (36.8) | 7,754 (36.9) | 3,280 (36.6) | 1.004 (0.944, 1.069) |
| 2-3 times / week | 61.89 (20.6) | 3,782 (18.0) | 2,407 (26.8) | 1.032 (0.956, 1.114) |
| 4-6 times / week | 921 (3.1) | 471 (2.2) | 450 (5.0) | 1.091 (0.919, 1.295) |
| 1 time / day | 462 (1.5) | 251 (1.2) | 211 (2.4) | 1.137 (0.896, 1.444) |
| 2 or more times / day | 229 (0.8) | 95 (0.5) | 134 (1.5) | 1.152 (0.797, 1.665) |
| Daily dietary salt intake, n (%) | | | |  |
| ＜4 g | 4,724 (15.8) | 3,637 (17.3) | 1,087 (12.1) | Reference |
| 4-6 g | 23,630 (78.8) | 16,480 (78.5) | 7,150 (79.7) | 1.055 (0.979, 1.137) |
| ＞6 g | 1,617 (5.4) | 885 (4.2) | 732 (8.2) | 1.047 (0.908, 1.208) |
| Frequency of seafood intake, n (%) | | | |  |
| Hardly ever | 3,612 (12.1) | 2,331 (11.1) | 1,281 (14.3) | Reference |
| Less than 1 time / week | 8,923 (29.8) | 6,217 (29.6) | 2,706 (30.2) | 0.942 (0.857, 1.035) |
| 2-3 times / week | 14,363 (47.9) | 10,312 (49.1) | 4 051 (45.2) | 0.999 (0.913, 1.094) |
| 4-6 times / week | 2,232 (7.4) | 1,612 (7.7) | 620 (6.9) | 1.025 (0.900, 1.166) |
| Once a day | 586 (2.0) | 395 (1.9) | 191 (2.1) | 1.022 (0.825, 1.268) |
| More than 2 times a day | 255 (0.9) | 135 (0.6) | 120 (1.3) | 0.893 (0.638, 1.250) |
| Nightlight, IQR | 52.63 (13.92, 83.44) | 52.63 (13.92, 83.44) | 52.63 (11.11, 59.14) | 0.999 (0.998, 1.000) |
| NDVI ^e^, IQR | 0.24 (0.21, 0.28) | 0.24 (0.21, 0.27) | 0.24 (0.21, 0.29) | 0.906 (0.526, 1.558) |
| PM_2.5_, IQR | 38.50 (37.25, 38.95) | 38.60 (37.50, 39.05) | 37.90 (37.00, 38.80) | 1.048 (1.035, 1.061) |
| NO_2,_ IQR | 36.45 (34.20, 37.50) | 36.85 (34.70, 37.80) | 35.20 (33.90, 36.90) | 1.177 (1.149, 1.206) |

BMI, body mass index; NDVI, normalized difference vegetation index; PM_2.5_, the particulate matter smaller than 2.5 micrometers; NO_2_, nitrogen dioxide.

a. BMI was missing in 890 cases in the primary school and 240 cases in the other school types.

b. Homework hours was missing in 238 cases in the primary school and 932 cases in the other school types.

c. Outdoor hours was missing in 1063 cases in the primary school and 376 cases in the other school types.

d. Sleep duration was missing in 334 cases in the primary school and 636 cases in the other school types.

e. NDVI was missing in 0 cases in the primary school and 46 cases in the other school types.

Table S4. Comparison of demographic and clinical characteristics between school myopia and high myopia.

| **Factors** | **Total myopia** | **School myopia** | **High myopia** | **OR (95%CI)** |
| --- | --- | --- | --- | --- |
|  | **(n=15,948)** | **(n=14,685)** | **(n=1,263)** |  |
| Sex, n (%) |  |  |  |  |
| Female | 8,027 (50.3) | 7,356 (50.1) | 671 (53.1) | Reference |
| Male | 7,921 (49.7) | 7,329 (49.9) | 592 (46.9) | 0.889 (0.770, 1.028) |
| Parental myopia, n (%) | | | |  |
| Neither | 5,108 (32.0) | 4,811 (32.8) | 297 (23.5) | Reference |
| Either | 10,840 (68.0) | 9,874 (67.2) | 966 (76.5) | 2.234 (1.887, 2.644) |
| BMI ^a^, IQR | 17.9 (15.6, 20.5) | 17.9 (15.6, 20.5) | 18.1 (15.4, 20.6) | 0.993 (0.974, 1.012) |
| Level of education, n (%) | | | |  |
| Primary school | 8,723 (54.7) | 8,496 (57.9) | 227 (18.0) | Reference |
| Middle school | 4,964 (31.1) | 4,452 (30.3) | 512 (40.5) | 4.868 (3.915, 6.054) |
| High school | 2,261 (14.2) | 1,737 (11.8) | 524 (41.5) | 12.066 (9.267, 15.710) |
| Mode of commuting to school, n (%) | | | |  |
| Non-motorized transport | 8,808 (55.2) | 8,221 (56.0) | 587 (46.5) | Reference |
| Private car | 5,506 (34.5) | 5,067 (34.5) | 439 (34.8) | 0.956 (0.818, 1.116) |
| Public transport | 1,411 (8.8) | 1,218 (8.3) | 193 (15.3) | 0.917 (0.726, 1.159) |
| Residential | 223 (1.4) | 179 (1.2) | 44 (3.5) | 1.150 (0.724, 1.826) |
| Seat rows, n (%) |  |  |  |  |
| 1-3 rows | 7,079 (44.4) | 6,536 (44.5) | 543 (43.0) | Reference |
| 4-6 rows | 6,910 (43.3) | 6,328 (43.1) | 582 (46.1) | 1.189 (1.028, 1.375) |
| 7-10 rows | 1,959 (12.3) | 1,821 (12.4) | 138 (10.9) | 0.991 (0.782, 1.257) |
| Time of homework ^b^, IQR | 1.0 (1.0, 2.0) | 1.0 (1.0, 2.0) | 2.0 (1.0, 2.0) | 0.981 (0.890, 1.081) |
| Time of outdoor activities ^c^, n (%) | | | |  |
| ＜1 hour | 4,860 (30.5) | 4,378 (29.8) | 482 (38.2) | Reference |
| 1-2 hours | 8,376 (52.5) | 7,750 (52.8) | 626 (49.6) | 1.003 (0.860, 1.171) |
| 2-3 hours | 2,028 (12.7) | 1,923 (13.1) | 105 (8.3) | 0.924 (0.716, 1.191) |
| Reading distance, n (%) | | | |  |
| Less than a foot | 3,608 (22.6) | 3,320 (22.6) | 288 (22.8) | Reference |
| Greater than a foot | 12,340 (77.4) | 11,365 (77.4) | 975 (77.2) | 0.957 (0.807, 1.135) |
| Rubbing eyes, n (%) | | | |  |
| Never | 1,808 (11.3) | 1,689 (11.5) | 119 (9.4) | Reference |
| Sometimes | 12,562 (78.8) | 11,573 (78.8) | 989 (78.3) | 0.991 (0.781, 1.256) |
| Frequent | 1,440 (9.0) | 1,299 (8.8) | 141 (11.2) | 1.108 (0.806, 1.523) |
| Always | 138 (0.9) | 124 (0.8) | 14 (1.1) | 0.729 (0.277, 1.916) |
| Ball games, n (%) | | | |  |
| Never | 4,765 (29.9) | 4,361 (29.7) | 404 (32.0) | Reference |
| 1 kind | 7,332 (46.0) | 6,745 (45.9) | 587 (46.5) | 1.051 (0.892, 1.238) |
| 2 kinds | 2,809 (17.6) | 2,613 (17.8) | 196 (15.5) | 0.987 (0.793, 1.228) |
| 3 kinds | 825 (5.2) | 773 (5.3) | 52 (4.1) | 1.007 (0.702, 1.444) |
| 4 kinds | 170 (1.1) | 150 (1.0) | 20 (1.6) | 2.487 (1.398, 4.423) |
| 5 kinds | 47 (0.3) | 43 (0.3) | 4 (0.3) | 0.941 (0.209, 4.237) |
| Sleep duration ^d^, IQR | 9.00 (8.33, 9.50) | 9.0 (8.5, 9.5) | 8.5 (7.8, 9.0) | 1.010 (0.914, 1.117) |
| Frequency of going to green spaces, n (%) | | | |  |
| Never | 848 (5.3) | 734 (5.0) | 114 (9.0) | Reference |
| Once every few month | 3,692 (23.2) | 3,344 (22.8) | 348 (27.6) | 0.870 (0.648, 1.167) |
| More than 1 time per month | 8,926 (56.0) | 8,283 (56.4) | 643 (50.9) | 0.727 (0.547, 0.967) |
| Almost every day | 2,482 (15.6) | 2,324 (15.8) | 158 (12.5) | 0.750 (0.536, 1.050) |
| Frequency of soft drinks, n (%) | | | |  |
| Hardly ever | 5,437 (34.1) | 5,055 (34.4) | 382 (30.2) | Reference |
| Less than 1 time / week | 5,920 (37.1) | 5,448 (37.1) | 472 (37.4) | 0.935 (0.790, 1.107) |
| 2-3 times / week | 3572 (22.4) | 3,256 (22.2) | 316 (25.0) | 0.958 (0.793, 1.157) |
| 4-6 times / week | 578 (3.6) | 525 (3.6) | 53 (4.2) | 0.852 (0.589, 1.233) |
| 1 time / day | 286 (1.8) | 259 (1.8) | 27 (2.1) | 1.104 (0.686, 1.776) |
| 2 or more times / day | 155 (1.0) | 142 (1.0) | 13 (1.0) | 0.744 (0.311, 1.782) |
| Daily dietary salt intake, n (%) | | | |  |
| ＜4 g | 2,284 (14.3) | 2,124 (14.5) | 160 (12.7) | Reference |
| 4-6 g | 12,648 (79.3) | 11,635 (79.2) | 1,013 (80.2) | 1.110 (0.896, 1.375) |
| ＞6 g | 1,016 (6.4) | 926 (6.3) | 90 (7.1) | 0.764 (0.534, 1.095) |
| Frequency of seafood intake, n (%) | | | |  |
| Hardly ever | 2,021 (12.7) | 1,830 (12.5) | 191 (15.1) | Reference |
| Less than 1 time / week | 4,761 (29.9) | 4,392 (29.9) | 369 (29.2) | 0.787 (0.630, 0.981) |
| 2-3 times / week | 7,542 (47.3) | 6,968 (47.4) | 574 (45.4) | 0.796 (0.646, 0.982) |
| 4-6 times / week | 1,164 (7.3) | 1,077 (7.3) | 87 (6.9) | 0.784 (0.568, 1.082) |
| Once a day | 314 (2.0) | 287 (2.0) | 27 (2.1) | 0.986 (0.606, 1.605) |
| More than 2 times a day | 146 (0.9) | 131 (0.9) | 15 (1.2) | 0.529 (0.220, 1.273) |
| Nightlight, IQR | 52.63 (13.92, 83.44) | 52.63 (13.92, 83.44) | 52.63 (13.92, 83.44) | 1.000 (0.997, 1.003) |
| NDVI ^e^, IQR | 0.24 (0.21, 0.28) | 0.24 (0.21, 0.28) | 0.23 (0.19, 0.29) | 1.196 (0.361, 3.970) |
| PM_2.5_, IQR | 38.55 (37.50, 39.00) | 38.55 (37.50, 39.00) | 38.55 (37.25, 39.00) | 1.025 (0.987, 1.064) |
| NO_2,_ IQR | 36.60 (34.50,37.50) | 36.60 (34.40, 37.55) | 36.60 (34.70, 37.30) | 0.988 (0.920, 1.062) |

BMI, body mass index; NDVI, normalized difference vegetation index; PM2.5, the particulate matter smaller than 2.5 micrometers; NO2, nitrogen dioxide.

a. BMI was missing in 464 cases in the school myopia and 39 cases in the high myopia.

b. Homework hours was missing in 705 cases in the school myopia and 162 cases in the high myopia.

c. Outdoor hours was missing in 634 cases in the school myopia and 50 cases in the high myopia.

d. Sleep duration was missing in 554 cases in the school myopia and 97 cases in the high myopia.

e. NDVI was missing in 32 cases in the school myopia and 11 cases in the high myopia.

**Table S5. The RMSEs and IOA of different machine learning models.**

|  | RMSE | IOA |
| --- | --- | --- |
| GBM | 0.30 | 0.59 |
| XGBOOST | 0.30 | 0.59 |
| XRT | 0.31 | 0.58 |

RMSE: the root mean squared error

IOA: Index of Agreement based on Willmott et al. (2011), which spans between -1 and +1 with values approaching +1 representing better model performance.

An IOA of 0.5, for example, indicates that the sum of the error-magnitudes is one half of the sum of the observed-deviation magnitudes. When IOA = 0.0, it signifies that the sum of the magnitudes of the errors and the sum of the observed-deviation magnitudes is equivalent. When IOA = -0.5, it indicates that the sum of the error-magnitudes is twice the sum of the perfect model-deviation and observed-deviation magnitudes. Values of IOA near -1.0 can mean that the model-estimated deviations about O are poor estimates of the observed deviations; but they also can mean that there simply is little observed variability - so some caution is needed when the IOA approaches -1.

Table S6. Odds ratios of myopia among all the participants.

| **Factors** | **Total** | **Myopia** | **Non-myopia** | **OR (95%CI)** |
| --- | --- | --- | --- | --- |
|  | **(N=29,971)** | **(n=15,948)** | **(n=14,023)** |  |
| Sex, n (%) |  |  |  |  |
| Female | 14,402 (48.1) | 8,027 (50.3) | 5,534 (39.4) | Reference |
| Male | 15,569 (51.9) | 7,921 (49.7) | 8,489 (54.5) | 0.788 (0.745,0.833) |
| Parental myopia, n (%) | | | |  |
| Neither | 10,642 (35.5) | 5,108 (32.0) | 5,534 (45.5) | Reference |
| Either | 19,329 (64.5) | 10,840 (68.0) | 7,648 (54.5) | 1.788 (1.685,1.897) |
| BMI ^a^, IQR | 17.6 (15.2, 20.3) | 17.9 (15.6, 20.5) | 17.1 (14.9, 20.0) | 1.035 (1.027,1.043) |
| Level of education, n (%) | | | |  |
| Primary school | 21,002 (70.1) | 8,723 (54.7) | 12,279 (87.6) | Reference |
| Middle school | 6,329 (21.1) | 4,964 (31.1) | 1,365 (9.7) | 6.291 (5.735,6.900) |
| High school | 2,640 (8.8) | 2,261 (14.2) | 379 (2.7) | 5.797 (4.899,6.860) |
| Mode of commuting to school, n (%) | | | |  |
| Non-motorized transport | 17,199 (57.4) | 8,808 (55.2) | 8,391 (59.8) | Reference |
| Private car | 10,362 (34.6) | 5,506 (34.5) | 4,856 (34.6) | 0.919 (0.868,0.974) |
| Public transport | 2,147 (7.2) | 1,411 (8.8) | 736 (5.2) | 1.032 (0.917,1.162) |
| Residential | 263 (0.9) | 223 (1.4) | 40 (0.3) | 1.895 (1.095,3.279) |
| Seat rows, n (%) |  |  |  |  |
| 1-3 rows | 13,098 (43.7) | 7,079 (44.4) | 6,019 (42.9) | Reference |
| 4-6 rows | 13,082 (43.6) | 6,910 (43.3) | 6,172 (44.0) | 0.923 (0.872,0.978) |
| 7-10 rows | 3,791 (12.6) | 1,959 (12.3) | 1,823 (13.1) | 0.863 (0.792,0.941) |
| Time of homework ^b^, IQR | 1.0 (1.0, 2.0) | 1.0 (1.0, 2.0) | 1.0 (1.0, 2.0) | 1.118 (1.073,1.164) |
| Time of outdoor activities ^c^, n (%) | | | |  |
| ＜1 hour | 7,994 (26.7) | 4,860 (30.5) | 3,134 (22.3) | Reference |
| 1-2 hours | 16,151 (53.9) | 8,376 (52.5) | 7,775 (55.4) | 0.956 (0.895,1.021) |
| 2-3 hours | 4,387 (14.6) | 2,028 (12.7) | 2,359 (16.8) | 0.921 (0.843,1.006) |
| Reading distance, n (%) | | | |  |
| Less than a foot | 6,208 (20.7) | 3,608 (22.6) | 2,600 (18.5) | Reference |
| Greater than a foot | 23,763 (79.3) | 12,340 (77.4) | 11,423 (81.5) | 0.885 (0.826,0.948) |
| Rubbing eyes, n (%) | | | |  |
| Never | 4,272 (14.3) | 1,808 (11.3) | 2,464 (17.6) | Reference |
| Sometimes | 23,227 (77.5) | 12,562 (78.8) | 10,665 (76.1) | 1.350 (1.248,1.460) |
| Frequent | 2,250 (7.5) | 1,440 (9.0) | 810 (5.8) | 1.762 (1.551,2.002) |
| Always | 222 (0.7) | 138 (0.9) | 84 (0.6) | 1.290 (0.896,1.858) |
| Ball games, n (%) | | | |  |
| Never | 8,570 (28.6) | 4,765 (29.9) | 3,805 (27.1) | Reference |
| 1 kind | 13,824 (46.1) | 7,332 (46.0) | 6,492 (46.3) | 0.981 (0.919,1.048) |
| 2 kinds | 5,519 (18.4) | 2,809 (17.6) | 2,710 (19.3) | 0.960 (0.884,1.043) |
| 3 kinds | 1,646 (5.5) | 825 (5.2) | 821 (5.9) | 1.041 (0.915,1.185) |
| 4 kinds | 331 (1.1) | 170 (1.1) | 161 (1.1) | 1.048 (0.799,1.375) |
| 5 kinds | 81 (0.3) | 47 (0.3) | 34 (0.2) | 1.044 (0.587,1.858) |
| Sleep duration ^d^, IQR | 8.50 (9.00, 9.67) | 9.00 (8.33, 9.50) | 9.45 (9.00, 9.83) | 0.928 (0.889,0.968) |
| Frequency of going to green spaces, n (%) | | | |  |
| Never | 1,309 (4.4) | 848 (5.3) | 461 (3.3) | Reference |
| Once every few month | 6,100 (20.4) | 3,692 (23.2) | 2,408 (17.2) | 1.069 (0.909,1.256) |
| More than 1 time per month | 17,203 (57.4) | 8,926 (56.0) | 8,277 (59.0) | 0.947 (0.810,1.106) |
| Almost every day | 5,359 (17.9) | 2,482 (15.6) | 2,877 (20.5) | 0.906 (0.768,1.069) |
| Frequency of soft drinks, n (%) | | | |  |
| Hardly ever | 11,136 (37.2) | 5,437 (34.1) | 5,699 (40.6) | Reference |
| Less than 1 time / week | 11,034 (36.8) | 5,920 (37.1) | 5,114 (36.5) | 1.004 (0.944,1.069) |
| 2-3 times / week | 61.89 (20.6) | 3572 (22.4) | 2617 (18.7) | 1.032 (0.956,1.114) |
| 4-6 times / week | 921 (3.1) | 578 (3.6) | 343 (2.4) | 1.091 (0.919,1.295) |
| 1 time / day | 462 (1.5) | 286 (1.8) | 176 (1.3) | 1.137 (0.896,1.444) |
| 2 or more times / day | 229 (0.8) | 155 (1.0) | 74 (0.5) | 1.152 (0.797,1.665) |
| Daily dietary salt intake, n (%) | | | |  |
| ＜4 g | 4,724 (15.8) | 2,284 (14.3) | 2,440 (17.4) | Reference |
| 4-6 g | 23,630 (78.8) | 12,648 (79.3) | 10,982 (78.3) | 1.055 (0.979,1.137) |
| ＞6 g | 1,617 (5.4) | 1,016 (6.4) | 601 (4.3) | 1.047 (0.908,1.208) |
| Frequency of seafood intake, n (%) | | | |  |
| Hardly ever | 3,612 (12.1) | 2,021 (12.7) | 1,591 (11.3) | Reference |
| Less than 1 time / week | 8,923 (29.8) | 4,761 (29.9) | 4,162 (29.7) | 0.942 (0.857,1.035) |
| 2-3 times / week | 14,363 (47.9) | 7,542 (47.3) | 6,821 (48.6) | 0.999 (0.913,1.094) |
| 4-6 times / week | 2,232 (7.4) | 1,164 (7.3) | 1,068 (7.6) | 1.025 (0.900,1.166) |
| Once a day | 586 (2.0) | 314 (2.0) | 272 (1.9) | 1.022 (0.825,1.268) |
| More than 2 times a day | 255 (0.9) | 146 (0.9) | 109 (0.8) | 0.893 (0.638,1.250) |
| Nightlight, IQR | 52.63 (13.92, 83.44) | 52.63 (13.92, 83.44) | 52.63 (13.92, 83.44) | 0.999 (0.998,1.000) |
| NDVI ^e^, IQR | 0.24 (0.21, 0.28) | 0.24 (0.21, 0.28) | 0.24 (0.21, 0.28) | 0.906 (0.526,1.558) |
| PM_2.5_, IQR | 38.50 (37.25, 38.95) | 38.55 (37.50, 39.00) | 38.45 (37.10, 38.90) | 1.048 (1.035,1.061) |
| NO_2,_ IQR | 36.45 (34.20, 37.50) | 36.60 (34.50,37.50) | 35.80 (33.90, 37.50) | 1.177 (1.149,1.206) |

BMI, body mass index; NDVI, normalized difference vegetation index; PM_2.5_, the particulate matter smaller than 2.5 micrometers; NO_2_, nitrogen dioxide.

a. BMI was missing in 503 cases in the myopia and 627 cases in the non-myopia.

b. Homework hours was missing in 867 cases in the myopia and 303 cases in the non-myopia.

c. Outdoor hours was missing in 684 cases in the myopia and 755 cases in the non-myopia.

d. Sleep duration was missing in 651 cases in the myopia and 319 cases in the non-myopia.

e. NDVI was missing in 43 cases in the myopia and 3 cases in the non-myopia.

**SI References**

1. Li, T., Wei, R., Du, B., Wu, Q., Yan, J., Meng, X., Liu, Y., Yang, Q., Kee, C. S., Huang, G., Yan, H, Prevalence of myopia among children and adolescents aged 6-16 during COVID-19 pandemic: a large-scale cross-sectional study in Tianjin, China. Br. J. Ophthalmol. 108, 879-883 (2024).
2. Flitcroft, D. I., He, M., Jonas, J. B., Jong, M., Naidoo, K., Ohno-Matsui, K., Rahi, J., Resnikoff, S., Vitale, S., Yannuzzi, L, IMI - Defining and Classifying Myopia: A Proposed Set of Standards for Clinical and Epidemiologic Studies. Invest. Ophthalmol. Vis. Sci. 60, M20-M30 (2019).
3. Wei, J., Li, Z., Chen, X., Li, C., Sun, Y., Wang, J., Lyapustin, A., Brasseur, G. P., Jiang, M., Sun, L., Wang, T., Jung, C. H., Qiu, B., Fang, C., Liu, X., Hao, J., Wang, Y., Zhan, M., Song, X., Liu, Y., Separating Daily 1 km PM2.5 Inorganic Chemical Composition in China since 2000 via Deep Learning Integrating Ground, Satellite, and Model Data. Environ. Sci. Technol. 57, 18282-18295 (2023).
4. Dadvand, P., Rivas, I., Basagaña, X., Alvarez-Pedrerol, M., Su, J., De Castro Pascual, M., Amato, F., Jerret, M., Querol, X., Sunyer, J., Nieuwenhuijsen, M. J., The association between greenness and traffic-related air pollution at schools. Sci. Total. Environ. 523, 59-63 (2015).
5. Elvidge, C. D., Zhizhin, M., Ghosh, T., Hsu, F. C., Taneja, J., Annual time series of global viirs nighttime lights derived from monthly averages: 2012 to 2019. Remote Sensing. 13, 922 (2021).
